# Tebentafusp elicits on-target cutaneous immune responses driven by cytotoxic T cells in uveal melanoma patients

**DOI:** 10.1172/JCI181464

**Published:** 2025-04-29

**Authors:** Ramon Staeger, Aizhan Tastanova, Adhideb Ghosh, Nicola Winkelbeiner, Prachi Shukla, Isabel Kolm, Patrick Turko, Adel Benlahrech, Jane Harper, Anna Broomfield, Antonio Camera, Marianna Ambrosio, Veronika Haunerdinger, Phil F. Cheng, Egle Ramelyte, James Pham, Stefanie Kreutmair, Burkhard Becher, Mitchell P. Levesque, Reinhard Dummer, Barbara Meier-Schiesser

**Affiliations:** 1 Department of Dermatology, University Hospital Zurich, Zurich, Switzerland.; 2 Medical Faculty, University of Zurich, Zurich, Switzerland.; 3 Functional Genomics Center Zurich, University of Zurich and Eidgenössische Technische Hochschule (ETH) Zurich, Zurich, Switzerland.; 4 Immunocore Ltd., Abingdon-on-Thames, United Kingdom.; 5 Department of Medical Oncology, The Kinghorn Cancer Centre, St. Vincent’s Hospital Sydney, Darlinghurst, New South Wales, Australia.; 6 School of Clinical Medicine, University of New South Wales Medicine and Health, Sydney, New South Wales, Australia.; 7 Institute of Experimental Immunology, University of Zurich, Zurich, Switzerland.; 8 Department of Medical Oncology and Hematology, University Hospital Zurich, Zurich, Switzerland.

**Keywords:** Dermatology, Oncology, Cancer immunotherapy, Melanoma, Skin

## Abstract

**BACKGROUND:**

Tebentafusp is the first T cell receptor–based bispecific protein approved for clinical use in HLA-A*02:01^+^ adult patients with unresectable/metastatic uveal melanoma. It redirects T cells toward gp100-expressing target cells, frequently inducing skin-related early adverse events.

**METHODS:**

This study investigated immunological and cellular responses using single-cell and spatial analysis of skin biopsies from patients with metastatic uveal melanoma treated with tebentafusp.

**RESULTS:**

81.8% of patients developed acute cutaneous adverse events, which correlated with improved survival. Multimodal analysis revealed a brisk infiltration of CD4^+^ and CD8^+^ T cells, while melanocyte numbers declined. Single-cell RNA-sequencing revealed T cell activation, proliferation, and IFN-γ/cytotoxic gene upregulation. CD8^+^ T cells colocalized with melanocytes and upregulated LAG3, suggesting potential for combination therapies with tebentafusp. Melanocytes upregulated antigen presentation and apoptotic pathways, while pigmentation gene expression decreased. However, gp100 remained stably expressed.

**CONCLUSION:**

Sequential skin biopsies enable in vivo pharmacodynamic modeling of tebentafusp, offering insights into immune activation, toxicity, and treatment response. Examining the on-target effects of bispecifics in tissues amenable to longitudinal sampling enhances our understanding of toxicity and therapeutic escape mechanisms, guiding strategies for treatment optimization.

**FUNDING:**

Cancer Research Foundation, Swiss National Science Foundation (323630_207029, 733 310030_170320, 310030_188450, CRSII5_183478), Iten-Kohaut Foundation, European Research Council no. 882424, University Priority Project Translational Cancer Research of the University of Zurich (UZH), UZH PostDoc grant (K-85810-02-01).

## Abstract 2

Tebentafusp is a bispecific protein that redirects T cells to target cells expressing the melanocyte-lineage antigen gp100 and has been approved for HLA-A*02:01+ adult patients with unresectable or metastatic uveal melanoma. . The majority of patients experience skin inflammation within hours after tebentafusp infusion, which is thought to be an on-target effect against normal melanocytes. This study investigated the immunostimulatory and -regulatory cellular responses to tebentafusp using single cell RNA-sequencing and multiplex-immunohistochemistry on sequential patient skin biopsies. The findings highlight the value of skin samples for pharmacodynamic modeling of anti-melanocytic bispecifics. An improved understanding of the on-target effects may reveal therapeutic opportunities such as potential synergies of tebentafusp with LAG3- or regulatory T cell-focused therapies.

## Introduction

Tebentafusp is approved for HLA-A*02:01^+^ adult patients with unresectable or metastatic uveal melanoma (mUM) and is the first T cell receptor–based (TCR-based) agent in clinical use (1). It is based on the ImmTAC (immune mobilizing monoclonal T cell receptors against cancer) platform (2) and targets the melanoma-associated antigen gp100 through a soluble TCR fused to an anti-CD3 T cell mobilizing domain (3). Unlike antibody-based treatments that are generally directed against membrane-bound proteins, the TCR-based bispecifics enable access to the vast pool of intracellular antigens as therapeutic targets (4).

The TCR is engineered for high-affinity binding of the gp100-derived 9-mer peptide YLEPGPVTV in the context of HLA-A*02:01 (5), the most common allele at this locus (6). gp100 Is a melanocyte-lineage antigen and plays an essential role in melanin pigment biosynthesis (7), with gp100 peptide-human leukocyte antigen (pHLA) complexes presented on the surface of normal melanocytes and on melanoma cells (8). Due to the high affinity of the TCR, cells with low target density on their surface are efficiently recognized and bound with a long half-life in a first step (4). The anti-CD3 single-chain variable fragment was optimized to have a lower affinity; therefore, T cell activation would follow pHLA recognition and not vice versa (2). Finally, prolonged engagement of the CD3 receptor on T cells induces polyclonal activation of T cells, irrespective of their cognate TCR specificity, resulting in release of IFN-γ and granzyme B (GZMB), which mediate target cell death dependent on gp100 pHLA abundance on the target cell surface (8, 9).

Uveal melanoma (UM) originates from melanocytes in the choroid or less commonly in the ciliary body or iris of the eye and frequently metastasizes to the liver (10). Approximately 50% of patients with UM develop metastatic disease, for which the prognosis is poor, with liver-directed therapies or systemic treatments with chemotherapies or immune checkpoint inhibitors (ICIs) showing limited survival benefits (11). In a pivotal phase III trial, despite relatively low objective response rates of 9%, treatment with tebentafusp resulted in a significantly longer overall survival (OS) compared with the investigators’ choice control group (the 1-year and 3-year OS rate in the tebentafusp arm was 73% and 27%, respectively, versus 59% and 18% in the control arm) (1, 12). As the first treatment to demonstrate a survival benefit in mUM, tebentafusp has become the new standard of care for patients with the HLA-A*02:01 allele.

Notably, the majority of patients treated with tebentafusp in the phase III trial developed cutaneous adverse events (cAE) such as “rash” (83%), pruritus (69%), and pigmentation disorders (45%) (1, 13). “Rash” was used as a composite term for a list of cAEs, including erythematous, maculopapular, and vesicular eruptions. These were mostly low grade, with none resulting in discontinuation of tebentafusp treatment, and showed a very early onset (1, 13). cAEs are likely an off-tumor/on-target effect from tebentafusp-mediated recruitment of T cells to gp100-expressing melanocytes in the skin (14). We reasoned that sequential skin biopsies may serve as an in vivo pharmacodynamic model to study tebentafusp-induced responses such as T cell activation, effects on target cells, and contributions of bystander cells. Given that cutaneous inflammatory responses against melanocytes may mirror processes in the tumor microenvironment under tebentafusp, research on cAEs could offer insights into the mechanisms of action and treatment resistance associated with TCR-based bispecifics.

## Results

**Figure 1:**
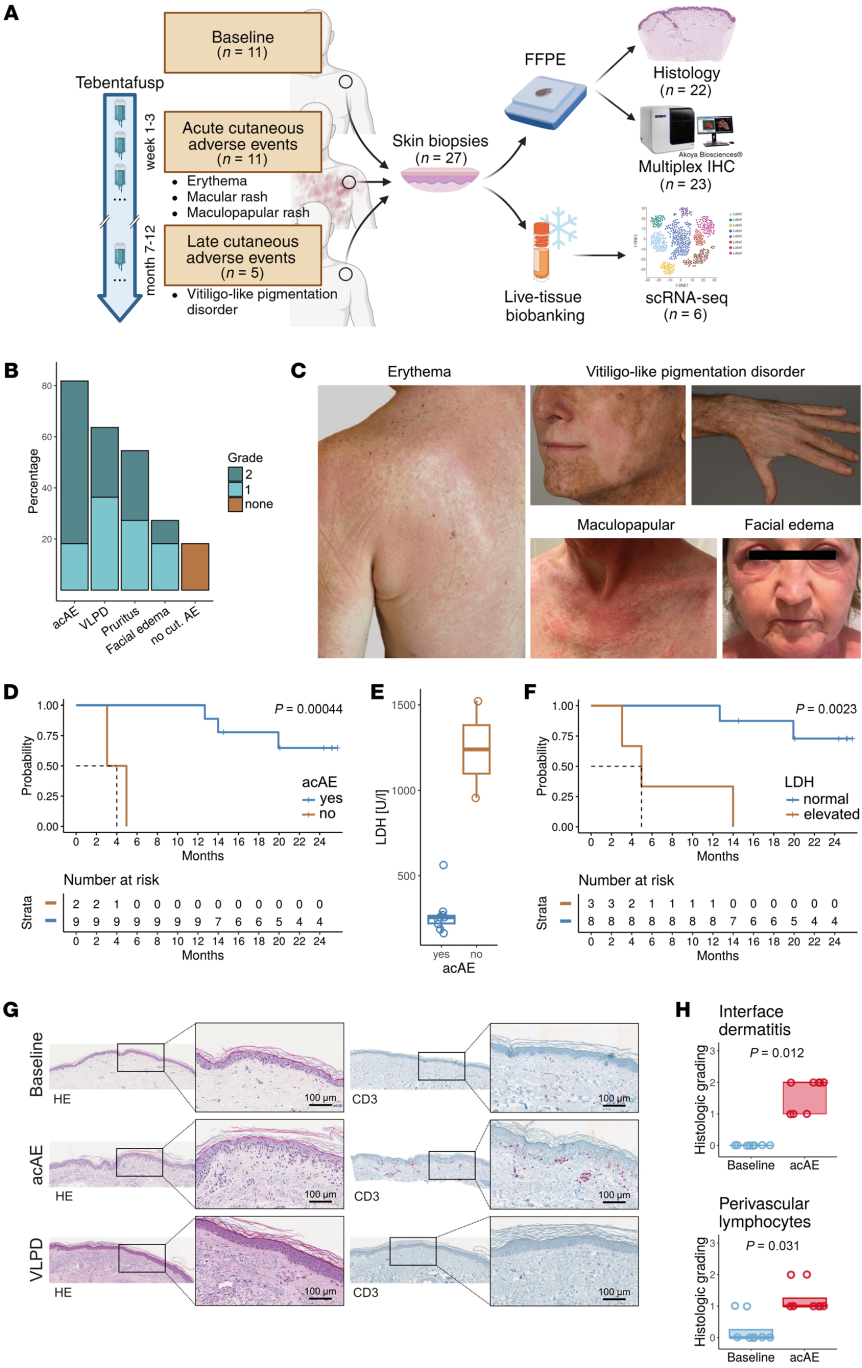
Study overview and characterization of clinical cohort. (**A**) Overview of experimental design (created with BioRender). (**B**) Incidence/grading of cAEs (*n* = 11). Grading according to CTCAE v5. (**C**) Representative clinical photographs of cAE observed under tebentafusp. (**D**) Kaplan-Meier curve of OS grouped by acAE development (log-rank test). (**E**) Baseline LDH levels grouped by acAE development (*n* = 11). (**F**) Kaplan-Meier curve of OS grouped by baseline LDH levels (log-rank test). (**G**) Representative H&E and CD3 stainings of baseline, acAE, and VLPD samples. (**H**) Histologic grading of interface dermatitis and perivascular lymphocytes in baseline and acAE (8 patients, paired; Wilcoxon’s test).

In this study, the cellular and molecular dynamics of cAEs in patients with mUM receiving tebentafusp were analyzed. Skin biopsies were collected from 11 patients at baseline and at the onset of an acute cAE (acAE) on tebentafusp treatment (or from unaffected skin for patients with no acAE) (Figure 1A). Additional lesional skin samples from vitiligo-like pigmentation disorder (VLPD) were collected later on in treatment from 5 patients (Figure 1A). Using multiplex immunohistochemistry (mIHC) and single-cell RNA-Seq (scRNA-Seq), a comprehensive assessment of the cellular pharmacodynamics in the skin in response to tebentafusp was conducted.

### Tebentafusp causes acAEs

cAEs to tebentafusp occurred in 9 of 11 patients (81.8%), most commonly as acute skin eruption 12–48 hours after the first 3 infusions, presenting as diffuse erythematous sunburn-like (*n* = 7), macular (*n* = 1), or maculopapular (*n* = 1) manifestations of grades 1–2 (Figure 1, B and C, and Supplemental Table 1; supplemental material available online with this article; https://doi.org/10.1172/JCI181464DS1), in line with previous reports (1, 15). Skin eruptions were frequently accompanied by pruritus (*n* = 6, 54.4%) (Figure 1, B and C, and Supplemental Table 1). Facial edema and a single bulla were present in 3 (27.3%) and 1 patient, respectively (Figure 1, B and C, and Supplemental Table 1). In all cases, acAEs were transient, responsive to oral antihistamines and topical steroids, and resolved by the next infusion a week later, apart from occasional superficial desquamation. Regarding delayed cAEs, VLPD occurred in 7 patients (63.6%), with a median onset of 192 days (range 85–275 days) following tebentafusp initiation (Figure 1, B and C, and Supplemental Table 1). Notably, all instances of VLPD were preceded by an acAE. Cytokine-release syndrome (CRS) was diagnosed in 72.7% of cases, with 62.5% being grade 2 (per Common Terminology Criteria for Adverse Events [CTCAE] v5) (16) and responding well to intravenous fluids and antipyretic medication, while the remaining cases were grade 1. Three or higher adverse events were not reported, and no patient discontinued treatment due to toxicity.

### acAEs correlate with outcome

After a median follow-up duration of 24.4 months (range 14.7–26.2 months), median progression-free survival (PFS) was 2.2 months (96% CI: 2.0 to not reached) (Supplemental Figure 1A) and the 1-year OS rate was 81.8% (95% CI: 61.9 to 100), while median OS was not reached (Supplemental Figure 1B). Development of acAEs correlated with significantly longer OS (*P* = 0.0004) (Figure 1D). However, occurrence of acAEs correlated with baseline serum lactate dehydrogenase (LDH) levels, an important prognostic marker (Figure 1, E and F). In a multivariate Cox’s proportional hazards model controlling for LDH, age, and sex, acAE was not found to be an independent predictor of PFS or OS.

### Tebentafusp induces T cell infiltration into the dermo-epidermal junction

Baseline skin biopsies were collected from all patients (*n* = 11) prior to tebentafusp initiation. On-treatment biopsies were taken from acAE lesional skin (*n* = 9) or from clinically unaffected skin in cases without acAEs (*n* = 2). Blinded histological evaluation of paired baseline and lesional skin biopsies (8 patients) was assessed by a certified dermatopathologist (Figure 1, G and H). The presence of interface dermatitis, defined as infiltration of T cells along the dermo-epidermal junction, cytoplasmic vacuolization of the basal epidermal layer, and apoptotic keratinocytes was a constant finding in acAE samples (*P* = 0.012, compared with baseline) (Figure 1H) and was absent in non-acAE samples (Supplemental Figure 1, C and D). This supports the proposed mechanism of tebentafusp-induced skin inflammation via T cell recruitment against gp100^+^ melanocytes in the basal epidermis, leading to bystander keratinocyte damage (1, 17, 18). Furthermore, increased dermal T cells in a perivascular distribution were observed (*P* < 0.031) (Figure 1H). In summary, tebentafusp-induced acAEs involved T cell infiltration.

### CD4^+^ and CD8^+^ T cells increase and melanocytes decrease in lesional skin

**Figure 2:**
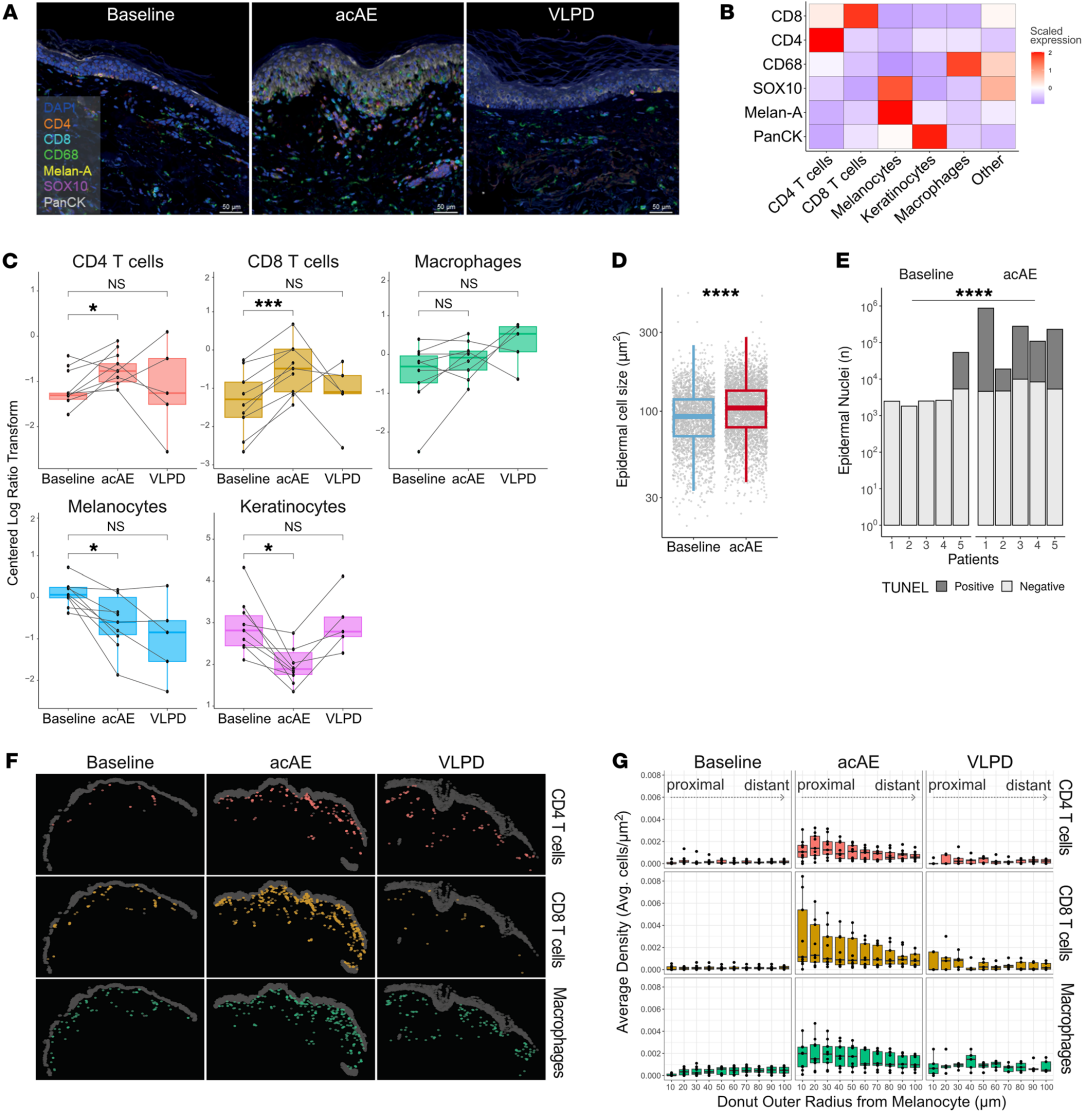
Spatial analysis of cutaneous inflammatory infiltrate on tebentafusp. (**A**) Representative mIHC scans of baseline, acAE, and VLPD skin samples. (**B**) Heatmap with scaled marker expression. (**C**) Cell-type composition at baseline (*n* = 9), acAE (*n* = 9), and VLPD (*n* = 5). Boxplots show the centered log ratio–transformed cell numbers (*t* test). (**D**) Epidermal cell sizes at baseline and acAE (*n* = 3, paired; Cohen’s *d* = 0.30). (**E**) Epidermal cell death at baseline and acAE skin samples, shown by TUNEL-positive and -negative epidermal nuclei (*n* = 5, paired). (**F**) Representative plot of the spatial distribution of macrophages, CD4^+^, and CD8^+^ T cells, relative to epidermis (gray) at baseline, acAE, and VLPD. (**G**) Spatial density of immune cells relative to melanocytes at baseline (*n* = 9), acAE (*n* = 9), and VLPD (*n* = 5), ranging from 0 μm (most proximal) to 100 μm (most distant) in 10 μm steps. *P < 0.05; ***P < 0.001; ****P < 0.0001.

Due to skin inflammation in tebentafusp-induced acAEs (Figure 1, B, C, G, and H), we investigated the composition, spatial distribution, and colocalization of the immune infiltrate. For this purpose, mIHC was performed on paired skin biopsies at baseline (*n* = 9), acAE onset (*n* = 9), and from VLPD (*n* = 5) (Figure 2A). Spectral unmixing and single-cell Leiden clustering detected 6 clusters that were annotated as CD4^+^ T cells, CD8^+^ T cells, CD68^+^ macrophages, pan-cytokeratin–positive (PanCK^+^) keratinocytes, and Melan-A^+^SOX10^+^ (cytoplasmic and nuclear markers) melanocytes (Figure 2, A and B).

Proportions of CD4^+^ and CD8^+^ T cells significantly increased in acAEs compared with baseline (adjusted *P* value [*P*-adj] = 0.026 and *P*-adj < 0.0003, respectively), while in VLPD lesions, T cell proportions were heterogeneous, with normalization in most patients but a further increase in a few patients (not significant) (Figure 2C). Macrophage proportions were not markedly altered in acAEs or VLPD compared with baseline (Figure 2C). Keratinocytes were significantly reduced in acAEs (*P*-adj = 0.017), as expected in the case of interface dermatitis with epidermal vacuolization (Figure 2C). Indeed, epidermal cell swelling in histology (*P* < 10^–15^, Cohen’s *d* = 0.3) (Figure 2D) and increased epidermal cell death marked by TUNEL staining (*P* < 10^–15^, odds ratio 12.2) were observed (Figure 2E and Supplemental Figure 1E) (19). Melanocyte proportions were decreased in acAEs (*P*-adj = 0.016) and remained below baseline levels in 4 out of 5 VLPD lesions (not significant) (Figure 2C). In summary, both CD4^+^ and CD8^+^ T cells increased in acAEs under tebentafusp, while melanocytes and keratinocytes decreased.

### CD8^+^ T cells are enriched in close proximity to melanocytes

Immune cell effector functions depend on spatial proximity to the target cell; for instance, CD8^+^ T cell cytotoxic activity requires cellular juxtaposition. Using mIHC, the coordinates and spatial relationships of the immune cells relative to the epidermis were mapped (Figure 2F). Next, as gp100^+^ cells are targeted by tebentafusp-mediated T cell redirection, the density of the immune cells was surveyed in incremental circles of 10 μm (1–2 cell widths) away from each melanocyte. In 4 of 9 patients with acAE, CD8^+^ T cells showed the highest enrichment in the immediate proximity of the melanocytes, in contrast to CD4^+^ T cells and macrophages, which were distributed more uniformly across skin tissue (Figure 2G). In VLPD skin, spatial proximities of all 3 cell types to melanocytes were reduced compared with acAEs, yet remained above baseline levels (Figure 2G). Thus, the CD8^+^ T cells preferentially localized and persisted in the immediate vicinity of the melanocytes, which is a prerequisite for tebentafusp-driven cytotoxic-effector functions.

### scRNA-Seq reveals T cell proliferation in acAE skin

To further investigate the cellular and molecular dynamics of tebentafusp-induced skin inflammation, scRNA-Seq was performed on paired baseline and acAE skin biopsies from 3 patients. After quality control (QC) filtering (Methods), a total of 23,638 high quality cells (mean 3,940 cells/sample) were available for downstream analysis. Ten major skin cell types were detected: keratinocytes (*KRT14*), melanocytes (*MITF*), lymphocytes (*CD2*), myeloid cells (*HLA-DRA*, *CD163*), fibroblasts (*COL1A1*), vascular endothelial cells (*CD93*), lymphatic endothelial cells (*FLT4*), pericytes (*PDGFRB*), smooth muscle cells (*ACTA2*), and glial cells (*MPZ*) (Supplemental Figure 2). The cell-type composition was comparable with previous findings in skin (20). Interestingly, glial cells showed significantly reduced abundance in acAEs compared with baseline (*P*-adj < 4 × 10^–5^).

**Figure 3:**
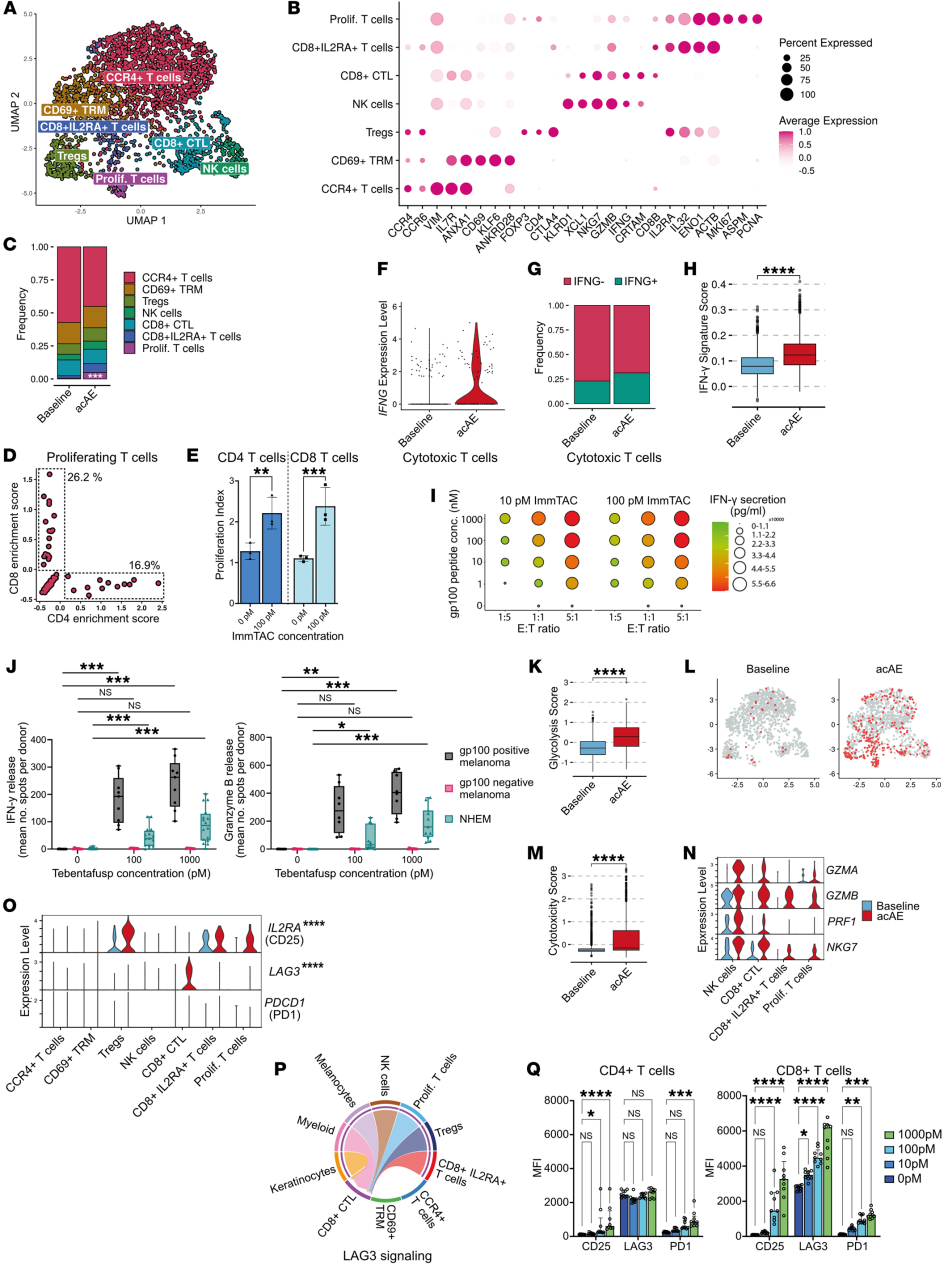
CTL activation and LAG3 upregulation in response to tebentafusp. (**A**) UMAP of T/NK cell subclusters in integrated baseline (1,343 cells) and acAE (1,175 cells) skin samples (*n* = 3, paired). (**B**) Marker gene dot plot and (**C**) cell-type composition bar plot (exact test). (**D**) Feature scatter plot showing the percentage of CD4^+^- and CD8A/CD8B-expressing cells. (**E**) Proliferation index of CD4^+^ and CD8^+^ T cells after coculturing with gp100^+^ cells, with/without gp100-ImmTAC (ANOVA). (**F**) Violin plot of IFNG expression. (**G**) Frequency of IFNG-positive CTLs. (**H**) Boxplot showing the IFN-γ gene signature (100) in T/NK cells (Wilcoxon’s rank-sum test). (**I**) Dotplot of IFN-γ protein concentrations in the supernatant of T cells cocultured with gp100 peptide–pulsed T2 cells in gp100-ImmTAC presence at different E:T ratios. (**J**) In vitro activity of tebentafusp against skin melanocytes. PBMCs and CD8^+^ T cells used as effector cells in IFN-γ and GZMB in ELISpot assays, respectively (*t* test). (**K**) Boxplot and (**L**) feature plot showing the glycolysis gene signature in T/NK cells (Wilcoxon’s rank-sum test). (**M**) Boxplot of the cytotoxicity signature in T/NK cells (Wilcoxon’s rank-sum test). (**N**) Violin plot showing the cytotoxic signature expression in T cell subclusters. (**O**) Violin plot showing IL2RA, LAG3, and PDCD1 in T/NK subclusters (Wilcoxon’s rank-sum test). (**P**) Chord diagram showing inferred LAG3 signaling in acAE between cell types. (**Q**) CD25, LAG3, and PD1 protein levels in CD4^+^ and CD8^+^ T cells after coculturing with gp100^+^ cell line at increasing gp100-ImmTAC concentrations (ANOVA). *P < 0.05; **P < 0.01; ***P < 0.001; ****P < 0.0001.

The mechanism of action of the gp100-ImmTAC molecule tebentafusp is based on recruitment of CD3^+^ T cells to gp100-expressing cells. gp100 is a melanocyte-lineage antigen expressed by epidermal melanocytes; hence acAE was suggested to be an on-target/off-tumor effect (1, 13). For an in-depth analysis of the lymphocyte cluster, second-level clustering was performed, which resulted in 7 subclusters of T and NK cells (Figure 3, A and B, and Supplemental Figure 3, A and B). The distribution of CD4^+^- and CD8^+^-expressing T cells is shown in Supplemental Figure 3C. Subcluster 1 was marked by *CCR4* and *CCR6*, both skin-homing chemokine receptors (21–23), as well as the tissue-residency–associated genes *VIM* and *ANXA1* (24, 25) (Figure 3B). This cluster contained both CD4^+^ and CD8^+^ T cells (Supplemental Figure 3D). High expression of *IL7R* indicated a naive/resting memory T cell phenotype (25–28). Subcluster 2 was marked by *CD69* and other markers of tissue-resident memory T cells (TRM) such as *KLF6*, *ANKRD28*, and *NR4A1* (24, 29–33) while *S1PR1* and *CCR7* were low (34, 35). Subcluster 3 Tregs were based on *FOXP3*, *CD4*, and *CTLA4* expression (36). Subclusters 4 and 5 were marked by cytotoxic gene expression and separated into NK cells based on *KLRD1*, *XCL1*, and *NKG7* (37, 38) and CD8^+^ cytotoxic T cells (CTL) based on *CD8A*, *IFNG*, *GZMA*, and *GZMB*. Subcluster 6 was marked by expression of *CD8A*, *CD8B*, and the activation-related markers *IL2RA*, *IL32*, *ENO1*, and *ACTB* (25, 39, 40), therefore corresponding to activated CD8^+^ T cells.

Subcluster 7 was proliferating T cells (*MKI67*, *ASPM*, *PCNA*) (25) (Figure 3B), the proportion of which increased more than 7-fold in acAEs (*P*-adj = 0.00057) (Figure 3C). This proliferating T cell cluster contained both CD4^+^ (16.9%) and CD8^+^ T cells (26.2%) (Figure 3D). Proliferation of both CD4^+^ and CD8^+^ T cells was replicated in vitro after coculturing with gp100-expressing cells in the presence of gp100-ImmTAC (Figure 3E). The proliferation of T cells in the skin on treatment suggests that colocalization with epidermal gp100-expressing cells (Figure 1G and Figure 2G) results in tebentafusp-mediated T cell activation in situ.

### IFN-γ and CTL activity are increased in acAEs

The Th1 cytokine (IFN-γ) is an important immunostimulatory and antitumor effector molecule. Increased systemic levels of IFN-γ were observed in patients within hours of tebentafusp infusion (3). In vitro, IFN-γ was predominantly secreted by CD8^+^ T cells in response to gp100-ImmTAC. In line with this, CD8^+^ CTLs upregulated *IFNG* in the skin on treatment, although this was not statistically significant (Figure 3F) (average log_2_ fold change [avg.log_2_FC] = 0.93). The frequency of IFN-γ–expressing CD8^+^ CTLs increased 1.4-fold (from 6.9% to 9.5%, not significant) (Figure 3G) and the IFN-γ gene expression signature increased significantly (*P*-adj = 2.6 × 10^–81^, Cohen’s *d* = 0.81) in acAEs (Figure 3H).

Normal melanocytes exhibit lower gp100 expression compared with melanoma (41). To explore the potential of low gp100 levels to activate T cell responses, mirroring the skin conditions, increasing concentrations of gp100 peptide were pulsed onto gp100-negative cells. IFN-γ (Figure 3I) secretion occurred at a very low gp100 peptide concentration of 1–10 nM, which likely represents the gp100 peptide range for healthy melanocytes (8, 42). IFN-γ secretion was gp100 level dependent, suggesting why lower T cell responses were observed against melanocytes compared with melanoma cells in vitro (8, 43). To investigate the relationship between gp100 levels and IFN-γ–mediated cytotoxicity, normal human epidermal melanocytes (NHEMs) were cocultured with effector cells at varying tebentafusp concentrations. gp100-positive and gp100-negative melanoma cell lines were used as positive and negative controls, respectively. Tebentafusp-dependent release of GZMB and IFN-γ was observed in cocultures with NHEMs (Figure 3J), with a more pronounced effect in gp100-positive melanoma cells. Higher response is likely due to 2-fold higher number of surface gp100-epitope counts in melanoma cells compared with NHEMs. Consistent with previous reports (43), no GZMB or IFN-γ release was detected in gp100-negative cells (Figure 3J). Together, these results demonstrate a gp100 level–dependent increase in IFN-γ activity in acAE skin upon gp100-ImmTAC treatment.

A strong overexpression of glycolysis genes across T subclusters (*P*-adj = 2.4 × 10^–115^, Cohen’s *d* = 0.99) (Figure 3, K and L) suggested a broad activation of T cells (44, 45), further supported by the marked downregulation of *IL7R* (avg.log_2_FC = –1.2, *P*-adj = 2.01 × 10^–80^) (Supplemental Figure 3E), a marker of naive T cell phenotypes that is downregulated following TCR stimulation (28, 46, 47). Upregulation of cytotoxic gene expression was observed (*P*-adj = 2.5 × 10^–62^, Cohen’s *d* = 0.72) (Figure 3M) in the CD8^+^ T cell subclusters, the NK cells, and the proliferating T cells (Figure 3N).

### CD8^+^ T cells upregulate LAG3 in acAEs

Besides T cell activation, immunoregulatory mechanisms were also observed in acAE skin. The α subdomain of the high-affinity IL-2 receptor *IL2RA* (CD25) is a marker of activated T cells (48) and was upregulated in the proliferating and the CD8^+^IL2RA^+^ T cells (*P*-adj=3.60 × 10^–11^) (Figure 3O). *IL2RA* is also implicated in immunoregulatory functions exerted by Tregs (49), where *IL2RA* was upregulated on tebentafusp treatment (Figure 3O). Furthermore, the immune checkpoint *LAG3* was overexpressed in acAEs, predominantly in CD8^+^ CTLs (*P*-adj = 2.1 × 10^–08^, respectively) (Figure 3O). Intercellular communication analysis through CellChat (50) revealed a strong activity of LAG3 signaling in CD8^+^ CTLs in acAEs (Figure 3P). In vitro, surface protein levels of CD25 and LAG3 significantly increased on T cells upon stimulation with gp100-ImmTAC in a coculture with gp100^+^ cells, validating the scRNA-Seq findings (Figure 3Q).

Interestingly, *PDCD1* was not expressed in acAEs (Figure 3O), but PD1 surface proteins were upregulated on T cells in vitro upon stimulation with gp100-ImmTAC (Figure 3Q). However, significant increases in PD1 were only observed at gp100-ImmTAC concentrations of 100 pM or 1,000 pM on CD8^+^ and CD4^+^ T cells, respectively, in contrast to LAG3, which was increased on CD8^+^ T cells at 10 pM (Figure 3Q). To validate the dynamics of LAG3 and PD1 in UM, a published bulk RNA dataset of melanoma metastases from patients treated with tebentafusp was analyzed (3). Paired baseline and on-treatment tumor samples were available from 2 patients with UM. Both showed an increase in *LAG3* expression, while *LAG3* was decreased in 6 of 11 cutaneous melanoma (CM) patients (Supplemental Figure 3F). Conversely, *PDCD1* was not detected in both UM patients either at baseline or on treatment, while it was expressed in 6 of 11 CM patients at both time points (Supplemental Figure 3G).

To assess the effectiveness of LAG3 inhibition in the context of tebentafusp treatment, CD8^+^ T cells were cocultured with target cells and treated with a combination of anti-LAG3 and anti-PD1 antibodies, as approved for CM (51), along with ImmTAC molecules (Supplemental Figure 3H). Significant activation of CD8^+^ T cells (CD69 upregulation) was observed following anti-LAG3/PD1 blockade (Supplemental Figure 3H). These findings, together with the observed increase in LAG3 expression in skin-infiltrating CD8^+^ T cells, suggest that LAG3/PD1 blockade — already established in clinical practice for CM (51) — may enhance ImmTAC-mediated T cell redirection against target cells.

### Melanocytes respond to IFN-γ, upregulate antigen presentation, and downregulate pigmentation genes

**Figure 4:**
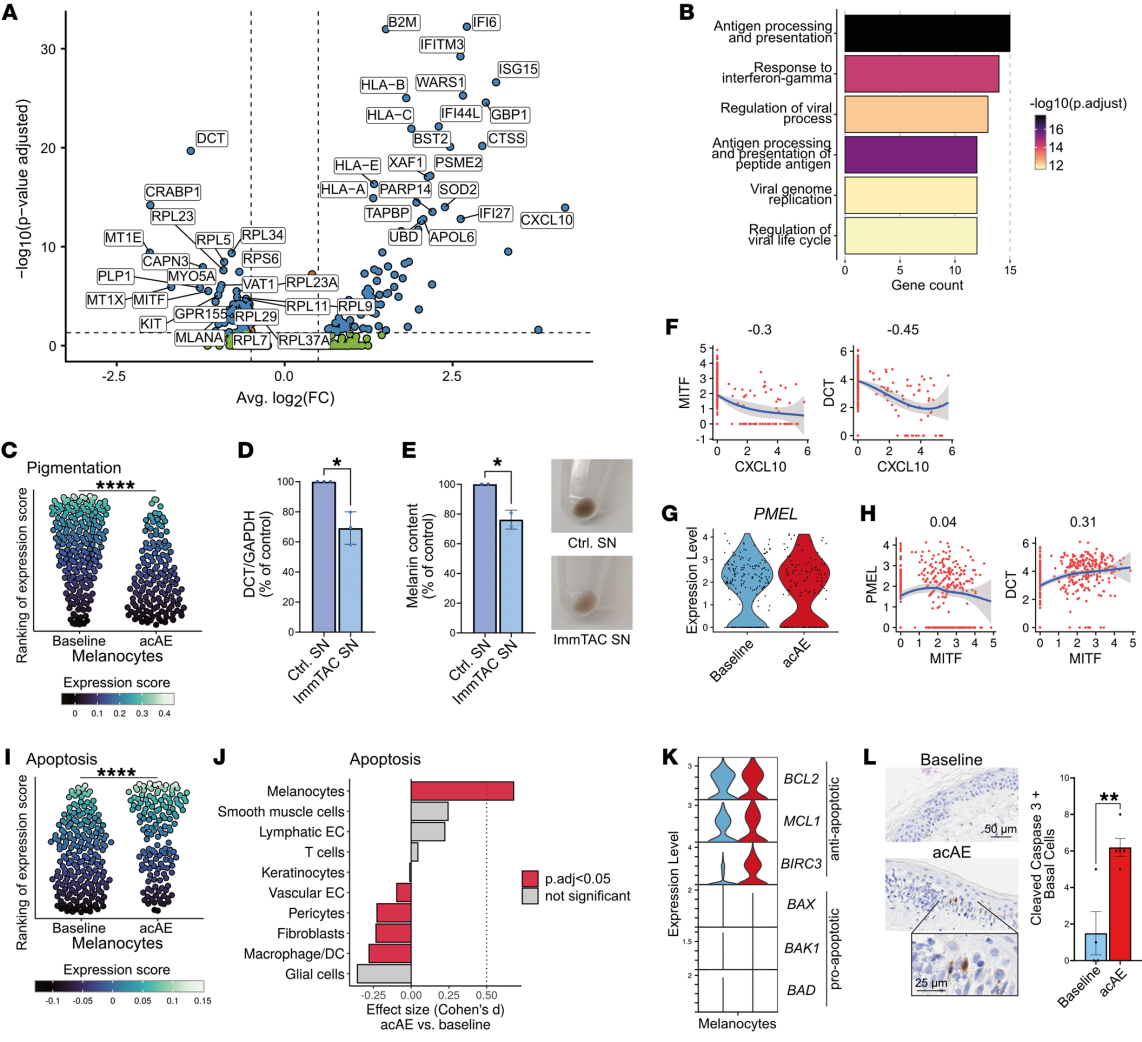
IFN-γ responses and apoptosis in melanocytes. (**A**) Volcano plot showing differential gene expression of melanocytes in acAE versus baseline skin samples (cut-offs: *P*-adj < 0.05, |log2FC| > 0.5) (**B**) GO pathway enrichment of upregulated genes in melanocytes of acAE versus baseline skin. (**C**) Bee-swarm plot showing the pigmentation gene signature (101) in melanocytes (Cohen’s *d* = 0.78) (Wilcoxon’s rank-sum test). (**D**) Normalized DCT protein levels in melanocytes treated with gp100-ImmTAC coculture supernatant versus control, quantified by WB (*n* = 3) (*t* test). (**E**) Melanin content of melanocytes treated with supernatant derived from gp100-ImmTAC coculture experiments versus control supernatant, quantified by photometric absorbance (*n* = 2) (*t* test). (**F**) Correlation of *MITF* and *DCT* expression with *CXCL10* in melanocytes (Pearson’s correlation). (**G**) PMEL expression in baseline and acAE melanocytes. Not significant. (**H**) Correlation of *MITF* with *PMEL* and *DCT* expression in melanocytes (Pearson’s correlation). (**I**) Bee-swarm plot showing the apoptosis gene signature (KEGG, in melanocytes) (Cohen’s *d* = 0.61) (Wilcoxon’s rank-sum test). (**J**) Barplot showing the effect size (Cohen’s *d*) and the direction of up- or downregulation of the apoptosis gene signature (102) in acAE versus baseline skin (Wilcoxon’s rank-sum test). (**K**) Violin plot showing the expression of anti- and proapoptotic genes in melanocytes. (**L**) Representative cleaved caspase-3 staining and quantification of positive cells in the basal epidermis (*n* = 5, paired) (*t* test). SN, supernatant. *P < 0.05; **P < 0.01; ***P < 0.001; ****P < 0.0001.

The melanocytes were of primary interest given their role as gp100-expressing cells in the normal skin. In the melanocytes of acAE samples, antigen processing and presentation (e.g., *B2M*, *TAPBP*, *HLA-A*, *HLA-B*, *HLA-C*, *HLA-E*) as well as response to IFN-γ (e.g., *CXCL10*, *IFI6*, *IFI27*, *IFITM3*, *PSME2*) Gene Ontology (GO) pathways were significantly overexpressed (Figure 4, A and B). Simultaneously, genes involved in melanin pigment synthesis (e.g., *DCT*, *MITF*) showed significant downregulation (*P*-adj = 2.5 × 10^–12^, Cohen’s *d* = 0.78) (Figure 4C).

Based on previous findings of IFN-γ–mediated downregulation of melanin synthesis (52), we hypothesized that tebentafusp-induced, immune cell–derived cytokines could be involved in the downregulation of melanin-associated genes. Indeed, decreased levels of DCT, a key enzyme in melanin synthesis, were found in melanocytes treated with conditioned supernatant (derived from gp100-ImmTAC–redirected PBMCs against gp100^+^ cells) in vitro (Figure 4D), which resulted in a visible reduction of melanin pigment (Figure 4E). DCT protein levels were rescued with an anti–IFN-γ, but not with an anti–IFN-β, antibody, suggesting the observed downregulation in pigmentation depends on IFN-γ (Supplemental Figure 4A). Furthermore, *DCT* and *MITF* expression were negatively correlated with *CXCL10* in melanocytes (Pearson’s correlation –0.45 and –0.3, respectively) (Figure 4F). These findings indicate that tebentafusp-induced, T cell–derived IFN-γ is involved in the activation of the melanocyte antigen presentation machinery and melanin synthesis inhibition.

### PMEL (gp100) is not downregulated in response to tebentafusp

In the melanocytes, *PMEL* (gp100) expression was not reduced on tebentafusp treatment, in contrast to other pigmentation-associated genes such as *DCT* and *MITF* (Figure 4, A and G). While both *DCT* and *PMEL* have previously been suggested to be regulated by *MITF* (53, 54), in our data only *DCT* but not *PMEL* expression was correlated with *MITF* (Pearson’s correlation 0.31 and 0.04, respectively) (Figure 4H). Furthermore, in contrast with *DCT* and *MITF*, *PMEL* expression did not negatively correlate with *CXCL10* (Figure 4F and Supplemental Figure 4B). However, the fraction of *PMEL*-expressing melanocytes showed a modest decrease from baseline to acAE of 7.0% (mean; range 1.5% to 15.9%) (Supplemental Figure 4C). These observations suggest *PMEL* expression to be more stable and less dependent on *MITF* and IFN-γ than other pigmentation genes.

### Apoptotic signaling in melanocytes specifically increases in acAEs

Next, we sought to explore melanocyte cell death as an explanation of the observed drop in melanocyte numbers (Figure 2C). There was a significant increase in the apoptosis-related gene expression signature in the melanocytes (Figure 4I), which was not detected in any other skin cell type (Figure 4J). Simultaneously, levels of antiapoptotic genes such as *BCL-2*, *MCL1*, and *BIRC-1* remained high (55) (Figure 4K). Indeed, quantification of apoptotic cells in acAE skin identified scattered cleaved caspase-3–positive cells, but with a significant increase compared with baseline skin (*P* < 0.01) (Figure 4L).

### Tebentafusp treatment induces a shift toward a proinflammatory macrophage state

**Figure 5:**
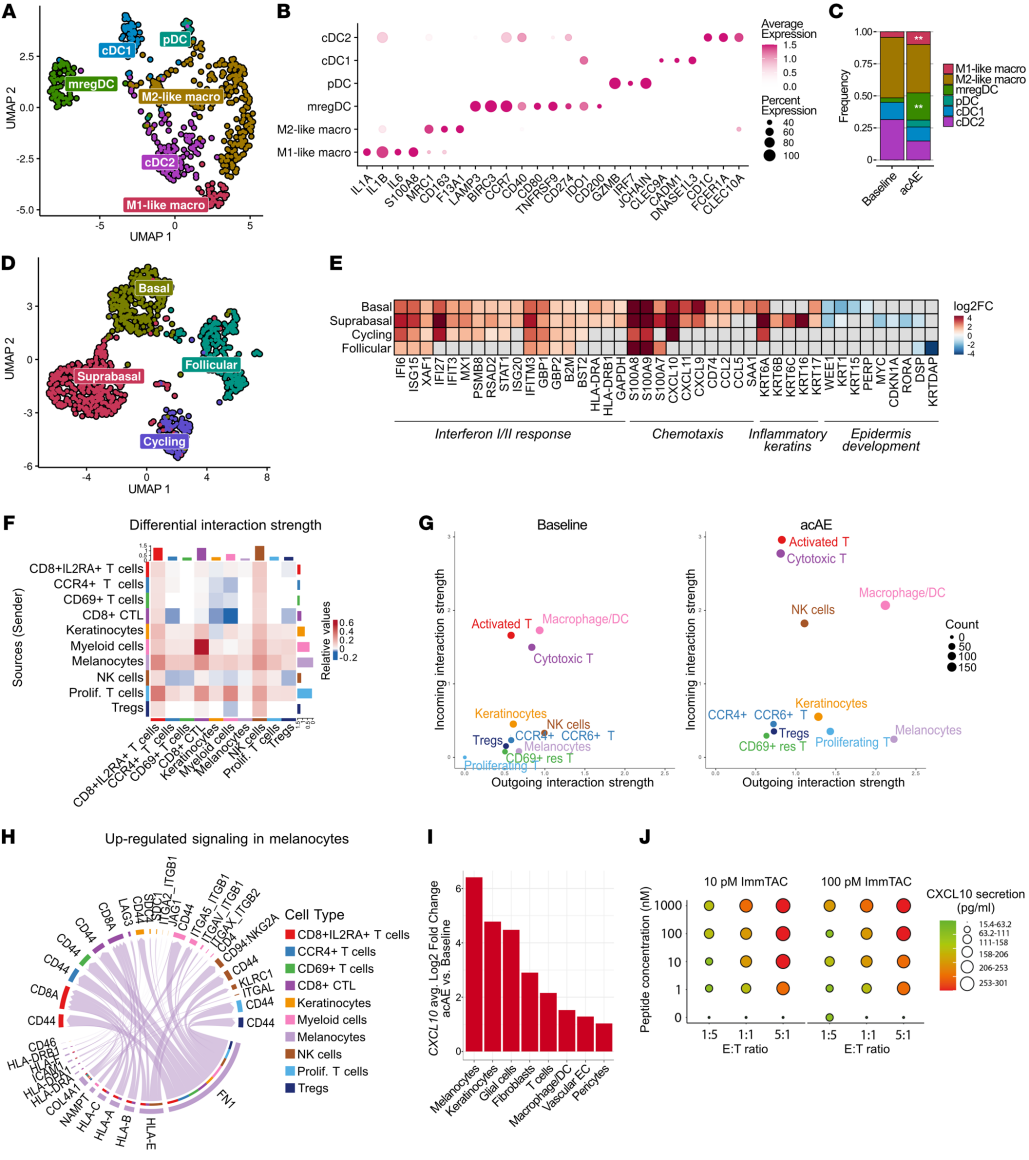
Network of proinflammatory and immunoregulatory functions in bystander cells. (**A**) UMAP of myeloid cell subclusters in integrated baseline (254 cells) and acAE (331 cells) skin samples (*n* = 3, paired). (**B**) Marker gene dotplot and (**C**) cell type composition bar plot of myeloid cell subclusters. Asterisks indicates significantly differentially abundant subclusters. (**D**) UMAP of keratinocyte subclusters in baseline and acAE skin samples (*n* = 3, paired). (**E**) Heatmap of differentially expressed genes in the keratinocytes of acAE versus baseline according to subcluster. Genes are grouped by biological function and significant differences are colored by log_2_ fold-change. (**F**) Heatmap of differential interaction strength in cell-cell communication between indicated cell types in acAE versus baseline skin (CellChat). (**G**) Outgoing and incoming signal strength according to cell type (CellChat). (**H**) Chord diagram showing the upregulated signaling pathways from melanocytes to other cell types (CellChat). (**I**) log_²_fold change of *CXCL10* expression in acAE versus baseline skin. (**J**) Dotplot of CXCL10 protein concentrations in the supernatant of T cells cocultured with gp100 peptide–pulsed T2 cells (gp100 ranging from 0–1000 nM) in gp100-ImmTAC presence (10/100 pM) at different E:T ratios, **P < 0.01.

Myeloid cells play key roles in cutaneous tissue repair and homeostasis (56). In the tumor microenvironment, they can assume pro- and antitumorigenic functions (57). Therefore, we aimed to elucidate their contribution to tebentafusp-mediated inflammation. Reclustering of myeloid cells resulted in 6 subclusters of macrophages and dendritic cells (DCs) (Figure 5, A and B). Macrophages separated into a proinflammatory M1-like (*IL1A*, *IL1B*, *IL6*) and an anti-inflammatory M2-like phenotype (*MRC1*, *CD163*, *F13A1*) (58) (Figure 5B). There was a significant increase in both the fraction of M1-like macrophages (*P* = 0.004) and the ratio of M1:M2 from baseline to acAE (OR 2.88, 95% CI: 1.39 to 5.96, *P* = 0.004) (Figure 5C and Supplemental Figure 5, A and B).

Furthermore, macrophages significantly upregulated the proinflammatory genes *S100A8* and *S100A9* (avg.log_2_FC > 3.29, *P*-adj < 6.44 × 10^–13^) (Supplemental Figure 5C). These are heterodimer-forming damage-associated molecular pattern (DAMP) molecules known to be released from myeloid cells during inflammation and to induce cytokine secretion and leukocyte recruitment (59). Similarly, expression of the extracellular matrix component versican (*VCAN*) was increased, which is involved in regulation of immune cell trafficking and activation (avg.log_2_FC = 1.85, *P*-adj = 6.81 × 10^–08^) (Supplemental Figure 5C) (60, 61). Together, these findings indicate a proinflammatory activation of macrophages in the skin on tebentafusp treatment.

### DCs with immunoregulatory functions accumulate on tebentafusp treatment

The DCs clustered into mature DCs enriched in immunoregulatory molecules (mregDC; *LAMP3*, *BIRC3*, *CCR7*) (62, 63), plasmacytoid DCs (pDC; *GZMB*, *IRF7*, *JCHAIN*) (64), classical DCs type 1 (cDC1; *CLEC9A*, *IDO1*, *CADM1*, *DNASE1L3*) (58), and classical DCs type 2 (cDC2; *CD1C*, *FCER1A*, *CLEC10A*) (58, 64) (Figure 5B).

The plasmacytoid dendritic cells (pDCs), which are specialized in type I interferon production, were only detected on treatment but not in baseline skin, consistent with previous observations of their recruitment to stressed skin (65) (Figure 5C and Supplemental Figure 5, A and B).

The mregDC subcluster significantly increased in acAEs (*P* < 0.005) in all patients (Figure 5C and Supplemental Figure 5A and B). These are migratory DCs with immunoregulatory functions, as evidenced by high levels of costimulatory genes such as *CD40*, *CD80* (B7-1), and *TNFRSF9* (4-1BB) as well as immunosuppressive genes *CD274* (PD-L1), *IDO1*, and *CD200* (Figure 5B) (66).

In summary, tebentafusp treatment caused cellular reorganization in the myeloid compartment. The recruitment of pDCs and mregDCs highlights the interplay of immunostimulatory and immunosuppressive functions induced by tebentafusp.

### Keratinocytes respond to tebentafusp treatment by upregulating proinflammatory genes

Besides barrier functions, keratinocytes can shape and amplify inflammatory signals in the skin (67). The keratinocytes subclustered into basal (*KRT5*), suprabasal (*KRT1*), cycling (*MKI67*), and hair follicle–associated clusters (*FOXC1*) (68–70) (Figure 5D and Supplemental Figure 5D). There was no significant change in the subtype composition from baseline to acAE skin across all patients (Supplemental Figure 5,E–G). IFN type I/II pathway responses and chemokine secretion were strongly upregulated (Figure 5E). Furthermore, the inflammatory intermediate filaments keratin 6 (*KRT6A*, *KRT6B*, *KRT6C*), 16 (*KRT16*), and 17 (*KRT17*), were upregulated, which are important regulators of epidermal innate immunity (Figure 5E) (71, 72). Several genes involved in epidermis development were downregulated, including *KRTDAP* (73), the transcription factor *MYC* (74), the nuclear hormone receptor *RORA* (75), and components of intercellular desmosome junctions *DSP* and *PERP*, as well as the cell-cycle inhibitors *CDKN1A* and *WEE1* (76) (Figure 5E).

### Intercellular communication increases in acAE lesional skin

To explore changes in cell-cell communication induced by tebentafusp, receptor-ligand activity was inferred through CellChat analysis (50). The inferred overall interaction strength nearly doubled from baseline to acAE skin (Supplemental Figure 5H). Intercellular communication was affected very broadly in acAEs as compared with baseline skin, with most cell types putatively interacting with each other (Figure 5F). On the receiving end, signaling to CD8^+^ T cells and NK cells saw the largest increase (Figure 5, F and G), largely by LAG3 signaling to CD8^+^ CTLs (Figure 3P).The strongest outgoing signal was observed in the melanocytes, followed by the proliferating T cells and the myeloid cells (Figure 5, F and G). In the melanocytes, fibronectin 1 (FN1) signaling and several HLA molecules were predicted as the most active pathways (Figure 5H).

### Melanocytes and keratinocytes secrete high levels of CXCL10 in lesional skin of tebentafusp-treated patients

Transient systemic increases of CXCL10 were reported as an acute response in patients treated with tebentafusp (3). Pharmacological modeling of cytokine dynamics following tebentafusp treatment predicted skin to be the major contributor of CXCL10 release (77). Interestingly, in our scRNA-Seq analysis, we identified melanocytes and keratinocytes as the cell types with the largest overexpression of *CXCL10* upon tebentafusp treatment (Figure 5I). Tebentafusp-mediated CXCL10 secretion was validated in vitro at the protein level in a gp100-dependent fashion (Figure 5J), suggesting that melanocytes and keratinocytes likely contribute to the transient systemic cytokine increase.

## Discussion

We analyzed 11 UM patients treated with tebentafusp, 81% of whom developed an acAE within hours of infusion. The appearance of early onset acAE is in line with previous findings that reported incidences of more than 80% at any grade within the first 3–4 weekly infusions (1, 12). Most reactions were transient erythema, with one case each of maculopapular and bullous lesions, similar to prior reports (18). Later in treatment, 63.6% developed a VLPD of skin or hair, a higher incidence than the previously reported 45%–57% (13, 17). This may be due to increased treatment beyond progression in real-world settings or the small cohort size.

The majority of patients developed acAEs within 12 to 48 hours after the first 3 weekly infusions. This rapid onset contrasts with the delayed cAEs seen with ICI-like anti-CTLA4/PD1/PDL1 (78). Unlike ICIs, which inhibit immunosuppressive molecules on T cells in the skin, tebentafusp directly recruits and activates T cells toward target antigen-expressing cells, triggering an immediate polyclonal T cell response. This likely explains the rapid T cell migration into the skin and the early onset of acAEs. The transient nature of acAEs aligns with the short half-life of tebentafusp and the observed cytokine peak within 24 hours after dose (3), distinguishing them from ICI-induced cAE, which can persist even after treatment discontinuation. These observations suggest that the acAEs reflect tebentafusp’s pharmacodynamics and pharmacokinetics in an on-target, off-tumor fashion.

Phase I/II and III trials found a correlation of acAE occurrence with longer OS, which we confirm in our small real-world analysis (1, 3). However, this association was dependent on other known prognostic factors, such as LDH levels, Eastern Cooperative Oncology Group performance score, and metastatic burden (14). Therefore, acAEs likely reflect overall immune fitness and responsiveness to tebentafusp, rather than serving as an independent predictor of outcome. These findings support the notion that acAEs mirror the tebentafusp’s pharmacodynamics. Histological analyses of skin reactions to tebentafusp revealed CD4^+^ and CD8^+^ T cell infiltration with a high density close to melanocytes, resembling a lichenoid reaction pattern. Similarly, T cell numbers increased in tumor tissue on treatment (3). scRNA-Seq analysis showed proliferation and high metabolic activity of CD4^+^ and CD8^+^ T cells in acAEs, indicating polyclonal T cell recruitment and activation, consistent with previous findings (9). The preferential enrichment and retainment of CD8^+^ T cells near melanocytes suggests their potentially contributing to tebentafusp-induced depigmentation. CellChat, a tool for analyzing cellular communication via scRNA-Seq, models signaling dynamics through ligand-receptor interactions (50) and can determine interaction strength, directionality, and cell-type–specific communication changes. CellChat predicted an increase in signal reception within CTLs, while melanocytes showed the highest increase in outgoing signaling, further supporting a tebentafusp-driven interaction between these 2 populations.

We observed Treg activation in tebentafusp-induced acAEs via *IL2RA* upregulation, a common feature of bispecific T cell engagers (45). This suggests that combining tebentafusp with Treg-targeted therapies or IL-2 variants that preferentially expand effector over regulatory T cells (79), may boost efficacy, though possibly at the cost of tolerability.

In the myeloid compartment, macrophages shifted to a proinflammatory M1-like phenotype likely due to the activation of skin-resident macrophages, as mIHC showed no significant increase in overall macrophage numbers. Overall macrophage levels remained unchanged, differing from other studies (14). However, mregDC levels increased significantly (*P* < 0.005) in acAEs. These immunoregulatory DCs are normally rare in skin (63), but quickly infiltrate inflamed sites (80). mregDCs exhibit both costimulatory (CD80) and immunosuppressive functions (PD-L1) (66), and their PD-L1 expression parallels its increase in tumors treated with tebentafusp, mostly driven by IFN-γ responses (3, 81, 82). Therefore, mregDCs may contribute to immunoregulatory signaling, though their role in tebentafusp activity remains unclear and warrants further investigation.

In patients treated with tebentafusp, CXCL10 showed the highest serum increase, peaking within 24 hours after dose (83). This CXCL10 surge correlated with extravasation of CXCR3^+^CD8^+^ T cells, leading to greater tumor shrinkage and improved survival (3, 83). In our study, melanocytes showed the greatest *CXCL10* increase among skin cells along with strong upregulation of IFN-γ response genes. This aligns with a pharmacodynamic model predicting that skin-resident immune cells are a major source of systemic cytokines (77). Furthermore, it suggests that melanocytes and keratinocytes contribute to CRS, warranting further research into CXCL10 and IFN-γ as potential CRS biomarkers or therapeutic targets.

Tebentafusp-induced T cell cytotoxicity and IFN-γ release triggered apoptotic signaling in melanocytes, resembling mechanisms seen in vitiligo (52). A study from Gellatly et al. identified cytotoxic CD8^+^ T cells as key mediators of melanocyte destruction and highlighted the CCL5/CCR5 axis in regulating CD8^+^ T cell and Treg interactions within the skin (84). Dysregulation of this pathway contributes to vitiligo progression, while IFN-γ amplifies inflammation, further driving melanocyte loss and pigment suppression. Similar to tebentafusp-induced changes, *CXCL10* and *IFNG* were highly expressed in keratinocytes and T cell populations, respectively (84). However, these conditions arise in distinct contexts: vitiligo is a chronic autoimmune disorder, while tebentafusp-induced skin inflammation is an acute, drug-induced response caused by on-target, off-tumor T cell activation. Notably, it was shown that patients who developed vitiligo under tebentafusp had higher survival rates, suggesting a potential link between off-tumor and on-tumor immune mechanisms (17).

While early on tebentafusp treatment there is a strong increase in CD8^+^ T cells, in the VLPD lesions, they were increased in only 2 patients. This is likely because biopsies were taken from the center of the lesion, a region characterized by lower disease activity than the borders, where depigmentation has already occurred and the T cells have left.

In our tebentafusp-treated cohort, melanocytes were significantly (*P*-adj = 0.016) reduced in acAE skin. While in vitro studies suggested lower gp100 levels in normal melanocytes limit direct tebentafusp-induced killing (43), cleaved caspase-3 staining indicated low apoptosis rates (85). This resistance may be due to high BCL-2 expression (55).

Intercellular communication analysis revealed a strong increase in paracrine signaling, especially among melanocytes, myeloid cells, and CD8^+^ T cells. Melanocytes upregulated FN1, an extracellular vesicle protein with antiapoptotic functions, linked to a mesenchymal melanoma phenotype and poor prognosis in CM and UM (86). Therefore, FN1’s role in tebentafusp resistance warrants further study.

Melanocytes in acAEs downregulated pigmentation genes, consistent with in vitro findings (8). Pigmentation loss correlated with CXCL10 expression, suggesting inflammation-driven pigment inhibition combined with scattered melanocyte death as the cause of VLPD. gp100 (*PMEL*) expression remained stable, independent of MITF or IFN-γ regulation. Immunohistochemistry showed no gp100 loss in melanoma metastases, and previously reported PMEL decreases were likely due to melanocyte loss rather than transcriptional downregulation (14). A 7% increase in PMEL-negative melanocytes in acAE suggests preferential killing of PMEL-expressing melanocytes.

In acAE, melanocytes upregulated antigen-presenting machinery genes, mirroring changes seen in tumor samples (3, 87). This machinery may have dual roles in tebentafusp treatment: it correlates with improved survival by increasing gp100-pHLA surface presentation, enhancing T cell activation (88, 89), but can also inhibit immune responses through HLA-A/B/C and HLA-E signaling to NK cell receptors (90) or HLA class II interactions with LAG3 (91). LAG3 expression was upregulated in acAE and UM metastases (*n* = 2) after tebentafusp, whereas PD1 showed variable expression, increasing in vitro at higher gp100-ImmTAC concentrations but not in skin or metastases, likely due to the use of healthy donor T cells in vitro. CellChat analysis and coculture assays confirmed LAG3 signaling in CTLs, aligning with prior findings that LAG3, rather than PD1, drives T cell exhaustion in UM (92, 93). Similar findings were reported with blinatumomab, another bispecific T cell engager, where LAG3 was upregulated but not PD1 (45). These results suggest that LAG3-targeted therapies could enhance tebentafusp efficacy, though potentially at the cost of increased toxicity.

We hypothesize that the on-target, off-tumor mechanisms observed in skin biopsies from tebentafusp-treated patients may provide valuable insights into its mechanism of action in the tumor microenvironment. However, intratumoral dynamics during treatment remain poorly understood due to the challenges of repeated liver metastasis sampling. For this reason, a direct comparison of matched skin and metastasis samples was not covered, representing a key limitation of our study.

In summary, we provide comprehensive insights into the single-cell dynamics associated with the on-target, off-tumor effects in skin inflammation in response to tebentafusp. Our key results of melanocytes and keratinocytes’ role in a feed-forward loop of cutaneous and systemic inflammatory processes, as well as the upregulation of LAG3 after treatment initiation, warrant a deeper investigation if these pharmacodynamics reflect the events that occur in the tumor microenvironment and possible therapeutic opportunities. Identifying shared or distinct targets that contribute to treatment escape or toxicity may lead to improved efficacy and tolerability of bispecific T cell engagers.

## Methods

### Sex as a biological variable

The patient cohort included both male and female patients, as detailed in Supplemental Table 1.

### Clinical information and survival analysis

We included consenting patients with mUM receiving tebentafusp (Kimmtrak) in an expanded access program at the University Hospital Zurich, Switzerland (ClinicalTrials.gov NCT04960891). Clinical information and experimental details are summarized in Supplemental Tables 1, 2 and 3. Adverse events were graded according to CTCAE v5. For survival analysis, R packages survival, version 3.5, and survminer, version 0.4.9, were used.

### LDH measurements

LDH was measured in the patients’ serum using the International Federation of Clinical Chemistry (IFCC) (https://diagnostics.roche.com/global/en/products/lab/ldhi2-cps-000156.html) method by Roche.

### Human primary tissue and live slow-frozen biobanking

Skin biopsies were collected from consenting patients and stored in the Dermatology Biobank as live slow-frozen samples for scRNA-Seq as previously described (20) and formalin-fixed paraffin-embedded (FFPE) samples for histology and immunohistochemistry (Supplemental Table 1). For histology and immunohistochemistry, FFPE skin samples were stained with H&E for standard histology or using the following antibodies for immunohistochemistry: anti-CD3 (Roche, 2GV6), anti-MelanA (Roche, A103), anti-SOX10 (Cell Marque, EP268), anti-tyrosinase (Roche, T311). Immunohistochemistry staining was performed on a Ventana BenchMark Ultra (Roche) with the UltraView Universal Alkaline Phosphatase Red Detection Kit. TUNEL and cleaved caspase-3 stainings were performed by Sophistolab, Switzerland.

H&E histologies were assessed by an experienced dermatopathologist based on qualitative grading (grade 0 = absent, 1 = weak, 2 = moderate, 3 = strong). Image analysis of TUNEL and cleaved caspase-3 stainings was performed in QuPath software, version 0.3.0. Automatic estimation of stain vectors was performed using a representative area. To assess epidermal cell death via TUNEL staining, the epidermis was selected and positive cell detection was run using preset parameters and a single threshold for mean nuclear staining intensity. From the same images, epidermal cell sizes were exported. For statistical analysis of TUNEL staining and epidermal cell sizes, a generalized linear mixed-effects model was fit using the *lmer()* function from the lme4 package in R, expressing the positive fraction of cells or the cell size, respectively, as a function of time point, with patient identity as a random variable and the model was fit using a binomial link function. For analysis of apoptotic cells via cleaved caspase-3 staining, the basal layer was selected and positive cell detection was run using preset parameters and a single threshold for mean cellular staining intensity.

### mIHC

For mIHC, Opal technology was used (Akoya Biosciences, NEL871001KT). The following primary antibodies were used: anti-CD8 (Abcam, ab4055), anti-CD68 (Abcam, ab213363), anti-MelanA (Novus Biologicals, NBP1-30151), anti-CD4 (Leica Biosystems, 4B12), anti-Sox10 (Abcam, ab268113), and anti-PanCK (Santa Cruz Biotechnology Inc., sc-8018). Staining was performed on a Bond RXm (Leica Biosystems) following the manufacturer’s instructions. Scanning was performed on a PhenoImager HT (Akoya Biosciences).

Spectral unmixing and cell segmentation were performed with inForm, version 2.4.9, software. Cell segmentation data was imported to R using the Giotto package, version 2.0.0.998. Cell identities of CD4^+^ T cells, CD8^+^ T cells, CD68^+^ macrophages, PanCK^+^ keratinocytes, and SOX10^+^MelanA^+^ melanocytes was based on leiden clustering. One unannotated cluster negative for these 6 markers was labeled as “Other.” Cell percentages were calculated for each patient separated by condition. Percentages were then transformed to centered log ratios using the clr() function from the R package compositions, version 2.0-6, enabling the compositional data to be analyzed independently of the dependencies between the components. Significance was calculated using the t_test() function from R package rstatix using an FDR correction to obtain adjusted *P* values.

Spatial location data obtained from inForm, version 2.4.9, software was utilized to plot cell locations to create a visualization of the patient biopsies in 2D space. From the phenoptr package, version 0.3.2 (94), the function count_within() was used to calculate the average number of each immune cell type within a specified radius of the melanocytes. Ten radii from 10 to 100 μm were used in 10 μm increments. Areas for each donut were calculated by subtracting the previous increment’s area, forming rings except for the initial 10 μm circle. The average number of immune cells in each space was divided by their areas to determine cell density.

### Enzymatic dissociation of live slow-frozen skin biopsies for scRNA-Seq

Enzymatic dissociation of live slow-frozen skin biopsies for scRNA-Seq was performed using 2-step digestion as previously described (95). Cell count and viability were accessed on a Luna-FL cell counter (Logos Biosystems, catalog L1001) using AOPI live/dead staining (Logos Biosystems, catalog F23001) with counting slides (Logos Biosystems, catalog L12005) and optimal cell concentration was adjusted according to 10X Genomics recommendations (700–1,200 cells/μl).

Single-cell processing was performed using a 10X Genomics Chromium Single-Cell Controller following the manufacturer’s guidelines. Paired-end sequencing (PE 28/8/0/91) was performed on the Illumina NovaSeq SP flow cell according to 10X Genomics recommendations, with more than 20,000 read pairs per cell for gene expression libraries.

### Data analysis of scRNA-Seq results

Raw reads were demultiplexed and aligned against the human reference genome assembly GRCh38.p13 using the 10x Genomics CellRanger, version 6.0.2, pipeline. The R package Seurat, version 4.1.1, was used for the downstream analyses of the filtered count matrices. Cells with unique feature counts of less than 250 or more than 4,000-6,000, unique UMI counts of more than 20,000, mitochondrial gene counts of more than 15%–30%, and ribosomal gene counts of more than 40% were discarded as part of QC. Filtered samples were log normalized and integrated using canonical correlation analysis. Integrated data were scaled and principal component analysis was performed using the top 2,000 variable features for dimensional reduction. Samples were clustered together using the Louvain algorithm with a resolution of 0.4 based on top 30 principal components (PCs). For each cell cluster, Wilcoxon’s rank-sum test was applied to identify the marker genes with log_2_ fold change greater than 0.25 and adjusted *P* value of less than 0.01 cut-offs. Cell clusters were annotated based on known markers from literature (20, 70, 96). For specific cell types, cells were reclustered using top 18 PCs and cluster resolution of 0.6 following the same steps as mentioned above.

Differential abundance analysis was performed for T and myeloid cell subcluster compositions. Exact test from the R package edgeR, version 4.0, was applied to measure the cell subcluster proportion differences between the 2 conditions.

Differential gene expression analysis (likelihood ratio test with patient effect as latent variable) was performed with the FindMarkers() function from the Seurat package. Differential genes with log_2_ fold-change greater than 0.5 and adjusted *P* value of less than 0.05 cut-offs were considered to be significant. GO pathway (GO BP) enrichment analysis was performed with the R package SCpubr, version 2.0.1 (97).

Gene expression signature scores were computed with the AddModuleScore() function from the Seurat package. Wilcoxon’s rank-sum test was used as a statistical test and effect size was calculated as Cohen’s *d* using the effect size package. The following signatures were used: gylcolysis: *ENO1*, *GAPDH*, *PGK1*, *PKM*, and *LDHA* (98); cytotoxicity: *GZMA*, *GZMB*, PRF1, *NKG7* (99); IFN-γ signaling: MSigDB hallmark gene set (100); pigmentation: GO:0043473 (101); apoptosis: Kyoto Encyclopedia of Genes and Genomes (KEGG) hsa04210, 87 genes (102).

Cell-cell interaction analysis was performed using the R package CellChat (50). The ligand-receptor database CellChatDB was updated with LAG3 receptor-ligand interactions (103). Only T cells, myeloid cells, keratinocytes, and melanocytes were considered for the CellChat analysis.

The R packages SCpubr, version 2.0.1, and Seurat, version 4.1.1, were used for visualization of scRNA-Seq results (97).

### Tumor expression data

From a published Nanostring tumor gene expression dataset of melanoma patients treated with tebentafusp (3), data for *LAG3* and *PDCD1*, which had not been reported on in that publication, was made accessible upon request. Results for 13 patients with a paired baseline and on-treatment sample (within 3 weeks post tebentafusp infusion) and information on melanoma subtype (2 uveal, 11 nonuveal) were available. The raw data was log_2_ normalized.

### In vitro assays

#### PBMC and T cell isolation.

100–200 ml blood was obtained from healthy donors and PBMCs were isolated by density gradient centrifugation over Lymphoprep (Axis-Shields). Negative T cell enrichment was performed using the Pan T-Cell Isolation Kit (Miltenyi) following the manufacturer’s instructions.

#### Measurement of T cell activation and proliferation in response to ImmTAC redirection.

MEL624 (obtained from NCI), an HLA-A*02:01^+^ gp100^+^ CM cell line, was used in ImmTAC redirection assays. For the T cell proliferation assay, pan T cells were prestained with 2.5 μM CellTrace Violet (CTV) (Thermo Fisher) according to the manufacturer’s guidelines. Tumor cells were cocultured with pan T cells (5:1 effector: target [E:T] ratio) in the presence or absence of gp100-ImmTAC concentrations of 10, 100, or 1,000 pM for 5 days. T cell activation was measured after 24 and 48 hours of redirection by flow cytometry. T cell proliferation was assessed at the end of the assay. Cells were harvested and stained with Zombie-NIR (Biolegend) to assess viability and fluorochrome-conjugated antibodies against CD3-APC (UCHT1), CD4-PE Cy7 (RPA-T4), CD8-BV650 (SK1), PD1-PE (NAT105), LAG3-BV785 (11C3C65; all from BioLegend), and CD25-BUV395 (2A3, BD Biosciences). Cells were fixed with BD Stabilizing Fixative (BD Biosciences). Samples were acquired on a BD LSRFortessaTM X-20 flow cytometer. Phenotypic markers of live CD4^+^ and CD8^+^ T cells were analyzed using FlowJo, version 10.5.3 (TreeStar, USA). T cell proliferation analysis (expansion index and precursor frequency) was determined based on CTV staining as previously described (104). To assess the influence of LAG-3 and PD-1 checkpoint blockade, T cells and tumor coculture assays in the presence of ImmTAC molecules were repeated in the presence or absence of anti-LAG3 (10 μg/ml, 11E3, Abcam) and anti–PD-1 (Pembrolizumab) Ab (10 μg/ml, Selleck Biotechnology Ltd.). Cells were then stained with antibodies against CD4 BUV496 (SK3), CD8 BUV805 (SK1; both from BD Biosciences), CD69 BV711 (FN50), and CD3 PE-Fire810 (17A2; both from BioLegend) and analyzed by flow cytometry as described above.

#### Measurement of cytokine and chemokine production.

T2 cells (ATCC) were pulsed with increasing concentrations of gp100 peptide (YLEPGPVTV) for 2 hours at 37°C. Cells were washed and cocultured with pan T cells at 1:5, 1:1, or 5:1 E:T ratios in the presence or absence of 10–100 pM tebentafusp (Immunocore Ltd.). Cytokines and chemokines within culture supernatants collected at 24 hours or 48 hours were measured by electrochemiluminescence using a combination of MSD U-plex and R-plex kits (Meso Scale Discovery). The assays were performed in duplicate following the manufacturer’s protocols and analyzed using the MSD QuickPlex SQ120 Reader (Meso Scale Discovery). Data analysis was performed using MSD Discovery Workbench, version 4.0.12, software (Meso ScaleDiscovery).

#### Melanin synthesis analysis.

NHEMs were used to assess the effects of tebentafusp-induced inflammation on the melanin synthesis pathway. NHEM4 (PromoCell), NHEM9 (ATCC), and NHEM10 (Lonza) were cultured according to the suppliers’ instructions and recommended media. Supernatants from tebentafusp-redirected PBMCs against melanoma cells (MEL526, obtained from the National Cancer Institute [NCI]) were collected and transferred onto NHEM cells. Cells were cultured for 72 hours in the presence or absence of 10 μg/ml neutralizing antibodies against IFN-γ (B27), or IFN-β (IFNb/A1; both from Biolegend). Cells were harvested and their melanin content was quantified by absorbance at 405 nm using a Clariostar spectrophotometer (BMG Labtech) as previously described (105). A standard curve was generated using synthetic melanin (Sigma-Aldrich) dissolved in 1 N NaOH (0–500 μg/mL). NHEM cell pellets were lysed with RIPA buffer (Thermo Fisher) containing protease inhibitors (Sigma Aldrich) followed by boiling at 95°C. Proteins were quantified using the Pierce BCA Protein Assay Kit (Thermo Fisher) and loaded onto Bolt 4%–12% Bis-Tris Plus gels (ThermoFisher). Melanin synthesis proteins were quantified by Western blot (WB) using antibodies against human GAPDH (6C5, Millipore), tyrosinase (ab180753), TRP1 (ab3312), TRP2/DCT (ab180753), and MITF (ab12039; all from Abcam) and goat anti-rabbit IgG (Goat anti-rabbit IgG secondary (catalog 7074) and goat anti-mouse IgG secondary antibodies (catalog 7076) (Cell Signaling Technology). WBs were performed using the Li-COR system, and membranes were scanned on the LI-COR C-DiGit Blot Scanner (LI-COR).

#### IFN-γ and GZMB ELISpot assays.

The melanoma cell lines Mel526 (HLA-A*0201^+^ and gp100^+^) and A375 (HLA-A*0201^+^ and gp100^–^; both obtained from ATCC) were used as positive and negative controls, respectively, and were maintained in RPMI supplemented with 2 mM l-glutamine, 10% FCS, 50 units/mL penicillin, and 50 μg/mL streptomycin. The number of PBMCs added per well varied according to which PBMC preparation had been previously titrated on Mel526 cells in order to determine the number of effector cells required per well. Reactivity between donor PBMCs and NHEM melanocytes in the presence of varying IMCgp100 concentrations was assessed by IFN-γ and GZMB ELISpot following the manufacturer’s protocol (BD Biosciences).

### Statistics

*P* values of less than 0.05 were considered significant. Student’s *t* tests were 2-tailed, and ANOVAs were 2 way.

### Study approval

The collection and use of clinical material for research purposes was approved by the Cantonal Ethics Committee Zurich (BASEC PB.2018-00194, KEK2019-02150), and patient informed consent was obtained for all human primary material.

### Data availability

Values for all data points in graphs are reported in the Supporting Data Values file. The scRNA-Seq data is available from the NCBI’s Gene Expression Omnibus database (GEO GSE259383).

## Author contributions

RS, AT, ER, MPL, RD, and BMS conceived the project. RS, AT, NW, IK, A Benlahrech, JH, A Broomfield, AC, and BMS collected data. RS, AT, AG, PS, PT, VH, PFC, SK, BB, and BMS analyzed data. Visualization: RS, AT, and AG. MPL, RD, and BMS provided resources. RS, AT, AG, and BMS wrote the original draft of the manuscript. IK, A Benlahrech, VH, ER, JP, SK, BB, MPL, RD, and BMS reviewed and edited the manuscript. RS is first because he collected clinical material, performed experiments, data analysis and visualization. AT is second because she performed data analysis and visualization.

## Supplementary Material

Supplemental data

ICMJE disclosure forms

Unedited blot and gel images

Supporting data values

## Version 1 05/01/2025

In-Press Preview

## Version 2 06/16/2025

Electronic publication
