# Supplementary material for: Tebentafusp elicits on-target cutaneous immune responses driven by cytotoxic T cells in uveal melanoma patients
Source: J Clin Invest. 2025 Apr 29;135(12):e181464. doi: 10.1172/JCI181464 (PMC12165791; doi:10.1172/JCI181464)
Supplement: Supplemental data [file jci-135-181464-s295.pdf]

Supplementary Figures

Supplementary Figure 1

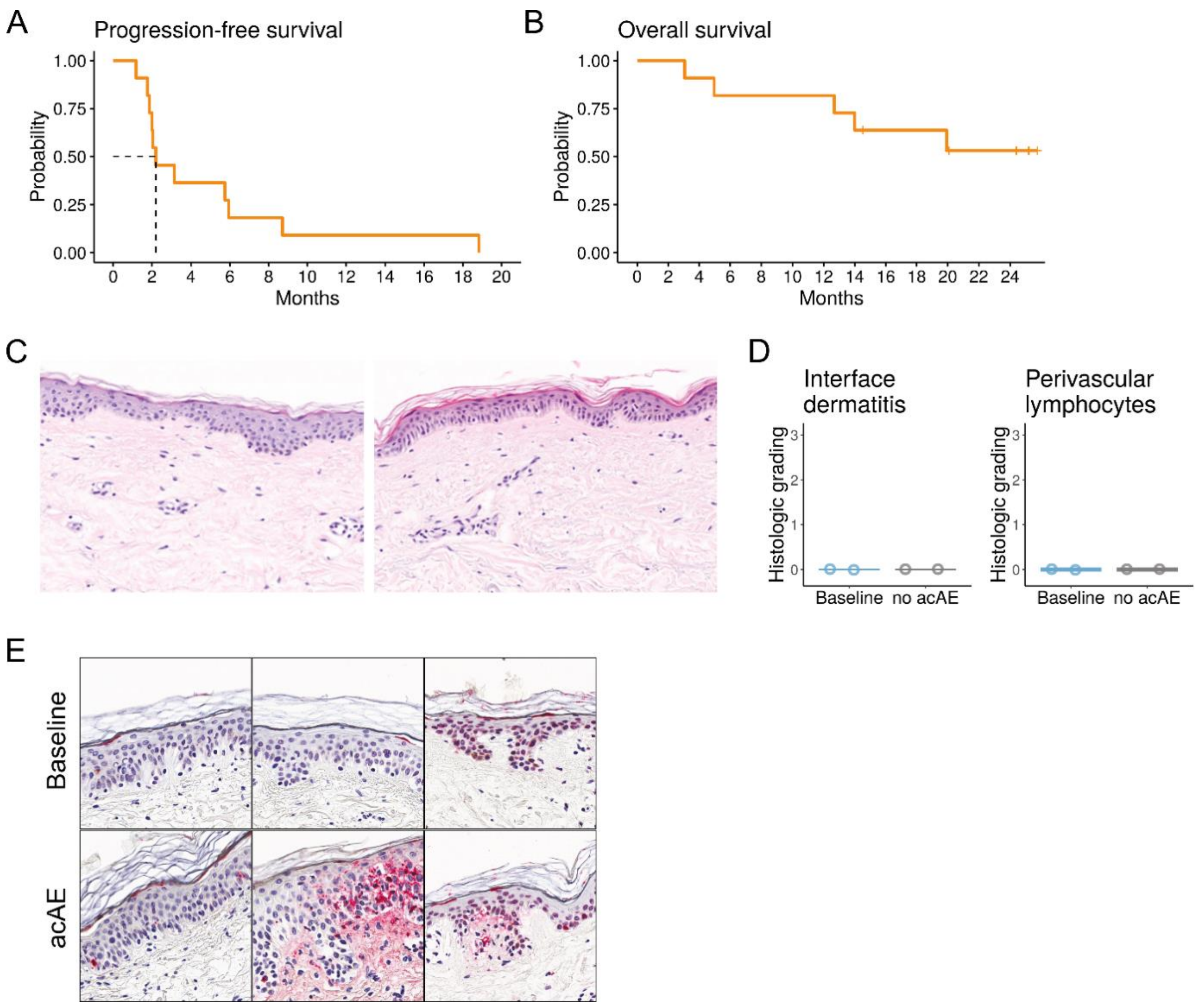

A) Kaplan-Meier curve of progression-free survival (PFS) and **B)** overall survival of patients with uveal melanoma treated with tebentafusp (n=11). Tick marks indicate censoring. **C)** Representative histology images of on-treatment skin biopsies from patients without acAE. **D)** Histologic grading of interface dermatitis and perivascular lymphocytes in baseline and non-acAE on-treatment skin samples (2 patients, paired). **E)** Representative TUNEL (Terminal deoxynucleotidyltransferase mediated dUTP Nick End Labeling) staining of baseline and acAE skin samples.

Supplementary Figure 2

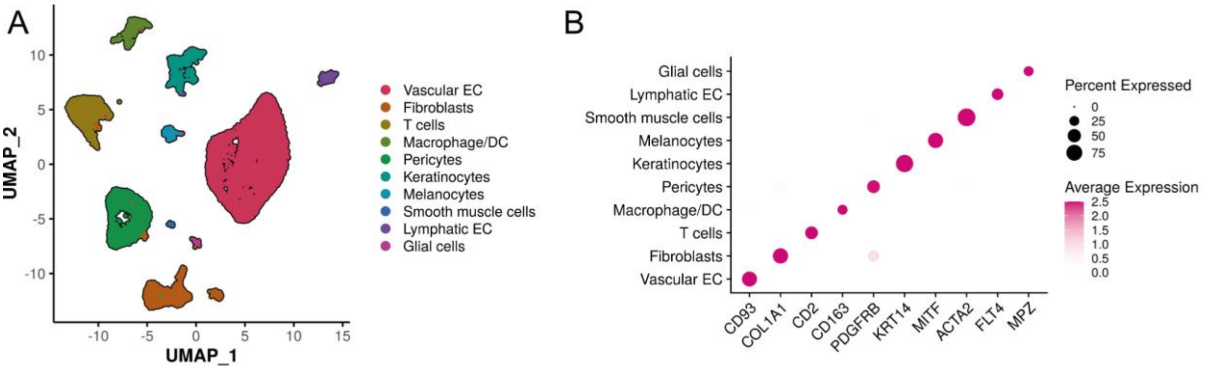

A) UMAP representation of all cell types in integrated baseline and acAE skin samples analyzed by single cell RNA-sequencing (3 patients, paired). **B)** Dotplot of lineage marker expression.

EC: Endothelial cells

Supplementary Figure 3

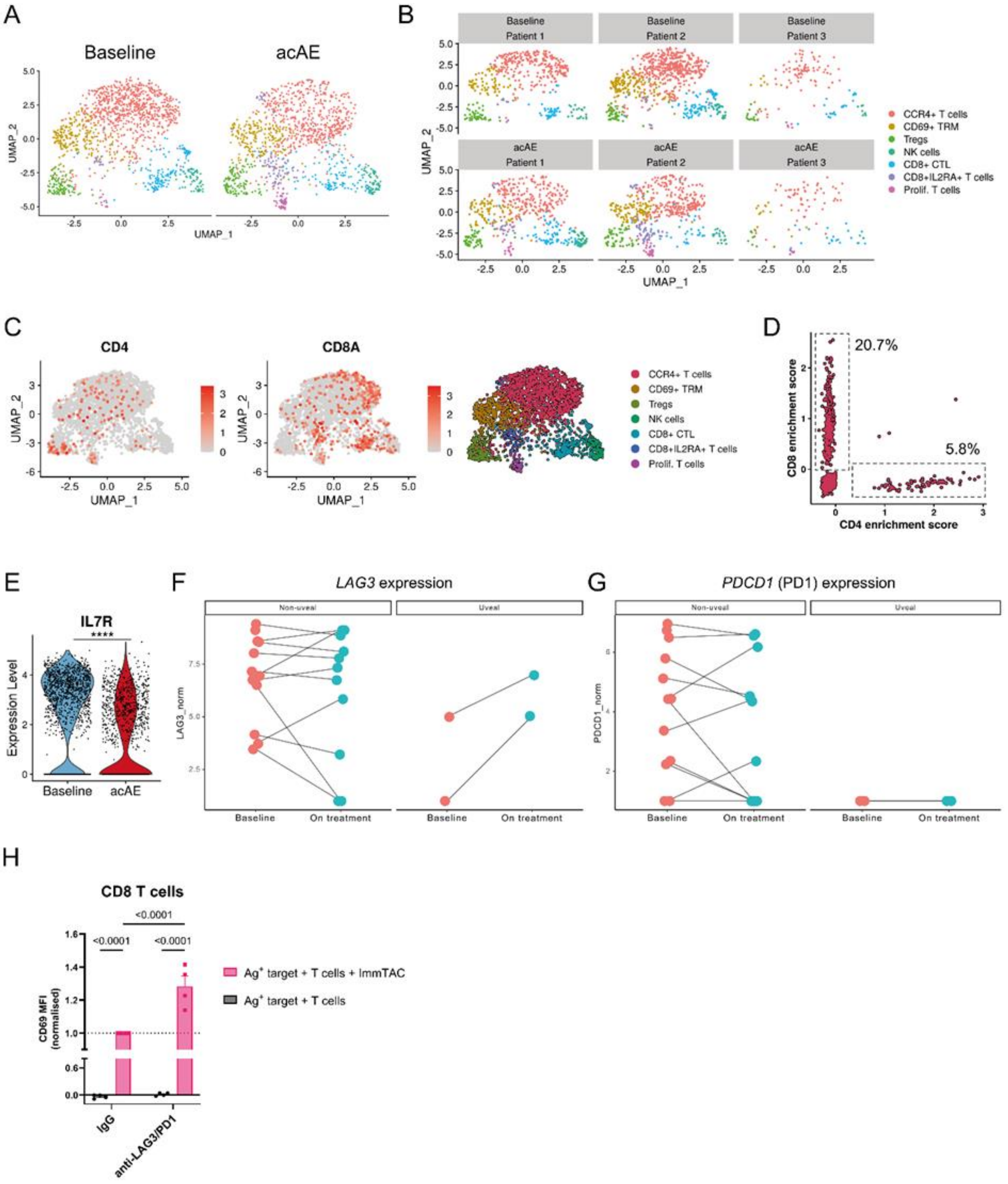

A) UMAP of T and NK cell subclusterss split by timepoint. **B)** UMAP split by timepoint and patient. **C)** UMAP feature plot showing CD4 and CD8A expression. **D)** Feature scatter plot showing the percentage of CD4 and CD8A/CD8B expressing cells in the CCR4+ T cell subcluster. **E)** Violin plot of IL7R

expression in T and NK cells on scRNA-seq. **F)** Log2-normalized expression of *LAG3* and **G)** *PDCD1* (PD1) in baseline and on treatment tumor biopsies from patients with uveal (n=2) or non-uveal (n=11) melanoma treated with tebentafusp, derived from a published RNA dataset (*Middleton, M.R., et al. (2020). Tebentafusp, A TCR/Anti-CD3 Bispecific Fusion Protein Targeting gp100, Potently Activated Antitumor Immune Responses in Patients with Metastatic Melanoma. Clin. Cancer Res. 26, 5869–5878.).* **H)** CD8 T cell activation as measured by CD69 when co-cultured with antigen expressing target cells with or without ImmTAC and anti-LAG3/anti-PD1 antibodies or IgG isotype control.

Ag: Antigen

Supplementary Figure 4

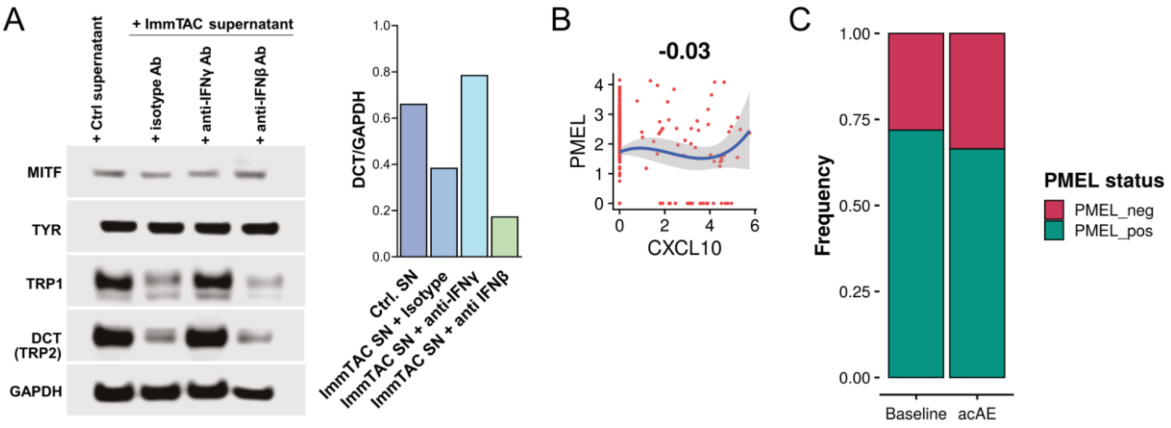

A) WB of the melanocytic proteins MITF (Melanocyte Inducing Transcription Factor), TYR (Tyrosinase), TRP1 (Tyrosinase Related Protein 1), and DCT (Dopachrome Tautomerase) derived from melanocytes treated with supernatant derived from gp100-ImmTAC co-culture experiments with the addition of an isotype control, anti-IFN-γ, or anti-IFN-β antibody. The normalized DCT values are shown as barplots. **B)** Correlation of *PMEL* and *CXCL10* expression levels in melanocytes on scRNA-seq. Pearson correlation is indicated. **C)** Frequency of *PMEL*-positive melanocytes on scRNA-seq.

Ctrl: Control, Ab: Antibody, ImmTAC: Immune Mobilizing Monoclonal T cell Receptor Against Cancer, GAPDH: Glyceraldehyde-3-Phosphate Dehydrogenase, SN: Supernatant, acAE: acute cutaneous adverse event

Supplementary Figure 5

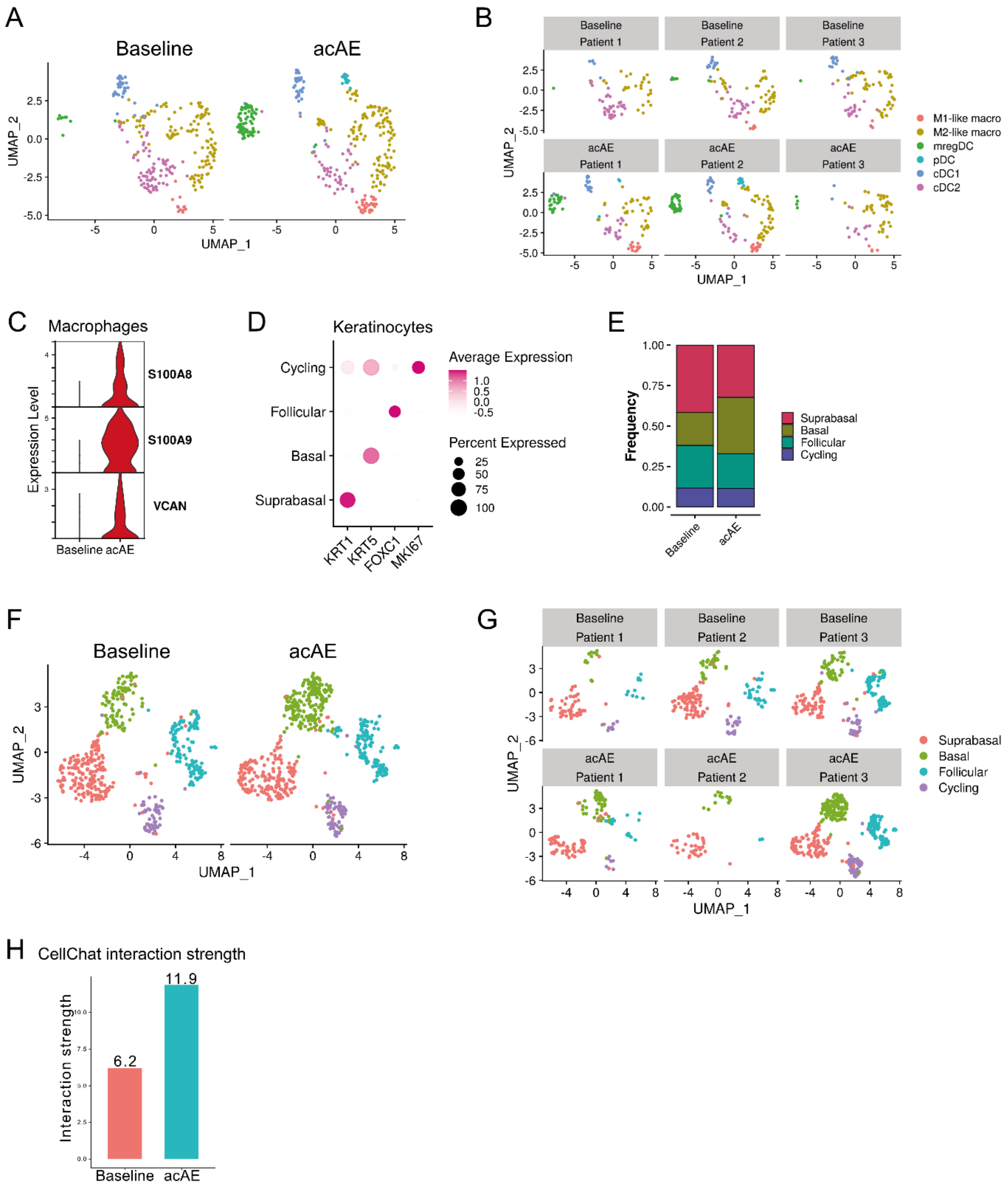

A) UMAP of myeloid cell subclusters split by timepoint and **B**) split by timepoint and patient. **C)** Violin plot of *S100A8*, *S100A9* and *VCAN* expression in macrophages on scRNA-seq. **D)** Marker gene dotplot and **E)** cell type composition barplot of keratinocyte subclusters on scRNA-seq. **F)** UMAP of keratinocyte subclusters split by timepoint and **G)** split by timepoint and patient. **H)** Cell-cell communication interaction strength as inferred from CellChat analysis.

acAE: acute cutaneous adverse event, cDC1: classical dendritic cell type 1, cDC2: classical dendritic cell type 2, mregDC: mature DC enriched in immunoregulatory molecules, pDC: plasmacytoid DC

Supplementary Table 1: Skin-related information

Patient	Sex	Age	Cutaneous adverse events	Follow-up skin biopsy	Biopsy from vitiligo-like pigmentation disorder	Assays
1	m	58	Diffuse erythema,<br>pruritus,<br>vitiligo-like pigmentation disorder	Day 15	Month 7<br><br>(tebentafusp treatment ongoing)	scRNA-seq<br>mIHC<br>histology
2	m	74	Diffuse erythema,<br>pruritus,<br>vitiligo-like pigmentation disorder	Day 8	Month 12<br><br>(tebentafusp treatment ongoing)	scRNA-seq<br>mIHC<br>histology
3	f	62	Diffuse erythema,<br>macular exanthema,<br>facial edema,<br>pruritus,<br>vitiligo-like pigmentation disorder	Day 2		scRNA-seq<br>mIHC<br>histology
4	m	57	Maculopapular exanthema,<br>partly diffuse erythema on face and neck,<br>vitiligo-like pigmentation disorder	Day 2	Month 8<br><br>(tebentafusp treatment ongoing)	mIHC<br>histology
5	m	66	Diffuse erythema,<br>pruritus,<br>vitiligo-like pigmentation disorder	Day 15	Month 9<br><br>(tebentafusp treatment ongoing)	mIHC<br>histology
6	m	56	Diffuse erythema,<br>vitiligo-like pigmentation disorder	Day 8	Month 7<br><br>(tebentafusp treatment ongoing)	mIHC<br>histology
7	f	47	Diffuse erythema,<br>single fluid-filled vesicle	Day 17		mIHC<br>histology
8	f	67	Diffuse erythema,<br>pruritus	Day 1		mIHC<br>histology
9	m	66	Diffuse erythema,<br>hand and facial edema, vitiligo-like pigmentation disorder	Day 8		mIHC
10	m	67	None	Day 14		histology
11	m	62	None	Day 8		histology

m: male, f: female, scRNA-seq: single cell RNA-sequencing, mIHC: multiplex immunohistochemistry

Supplementary Table 2: Tumor-related information

Patient #	Sex	Age	Uveal melanoma disease stage	Site of metastases	Previous treatments	Cytokine-release syndrome (CRS) on tebentafusp	Treatment of CRS
1	m	58	Stage IV	Lymph nodes, liver, bone, retroperitoneal space, pleura	Dacarbazine, Ipilimumab/Nivolumab, Temozolamid, Denosumab	CRS grade 2	fluids, paracetamol, metamizole, morphine
2	m	74	Stage IV	Liver	Ipilimumab/Nivolumab	CRS grade 2	fluids, paracetamol, metamizole
3	f	62	Stage IV	Liver, lung	Pembrolizumab, Temozolamid, Ipilimumab/Nivolumab	CRS grade 2	fluids, paracetamol, metamizole
4	m	57	Stage IV	Liver	none	CRS grade 2	fluids, paracetamol
5	m	66	Stage IV	Liver	Ipilimumab/Nivolumab	CRS grade 1	paracetamol, metamizole
6	m	56	Stage IV	Liver, lymph nodes	none	no CRS	none
7	f	47	Stage IV	Liver, bone	none	CRS grade 2	fluids, ibuprofen
8	f	67	Stage IV	Liver	none	no CRS	none
9	m	66	Stage IV	Liver	none	no CRS	none
10	m	67	Stage IV	Liver, brain, lymph node, pleura, pericardium, lung, muscle, thyroid, pancreas, colon, abdominal soft tissue, kidney, bone	Ipilimumab/Nivolumab, Temozolamid, Mekinist, Denosumab	CRS grade 1	paracetamol, metamizole
11	m	62	Stage IV	Liver, lung, lymph node, subcutaneous tissue, muscle	Ipilimumab/Nivolumab, Temozolamid, Pembrolizumab, Lenvatinib, Carboplatin	CRS grade 1	paracetamol, domperidone

CRS: Cytokine-release syndrome (according to CTCAE v5)

Supplementary Table 3: Vitiligo-like pigmentation disorder

Patient	Fitzpatrick Phototype	Distribution pattern of VLPD	Signs of activity (trichrome, confetti)	Diagnosis of VLPD	Biopsy timepoint	Biopsy site	VLPD dynamic at time of biopsy
1	IV	Generalized, symmetrical, mottled with confluent patches. Generalized leukotrichia.	confetti and trichrome	Month 3	Month 7	Upper arm	Expanding
2	III	Generalized, symmetrical, mottled. Generalized leukotrichia.	confetti	Month 9	Month 12	Lower arm	Stable
4	II	NA	confetti	Month 6	Month 8	Lower arm	Stable
5	III	Leukotrichia of eye brows and lashes.	confetti	Month 7	Month 9	Face	Expanding
6	II	Generalized, symmetrical, mottled with confluent patches. Generalized leukotrichia.	confetti	Month 5	Month 7	Face	Expanding

VLPD: vitiligo-like pigmentation disorder
