# Supplementary figures and images for: Tebentafusp elicits on-target cutaneous immune responses driven by cytotoxic T cells in uveal melanoma patients
Source: J Clin Invest. 2025 Apr 29;135(12):e181464. doi: 10.1172/JCI181464 (PMC12165791; doi:10.1172/JCI181464)

## Suppl. Figure 4A: Western Blot Original Images

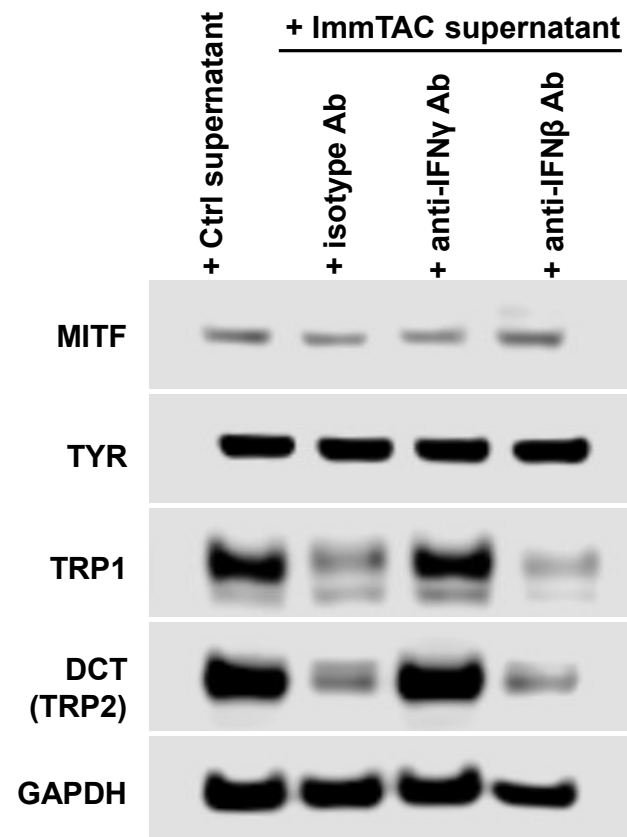

TYR

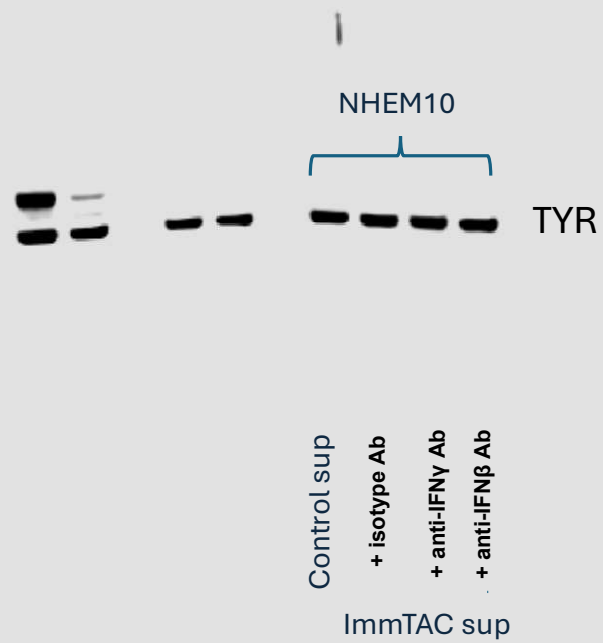

GAPDH

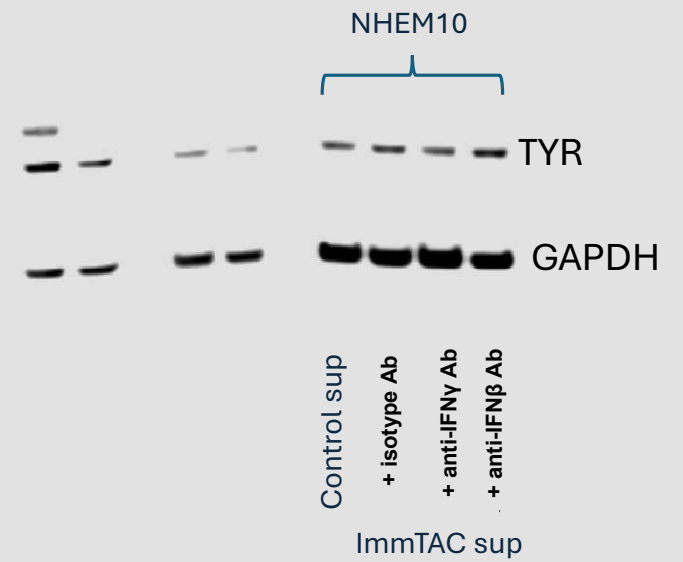

TRP1

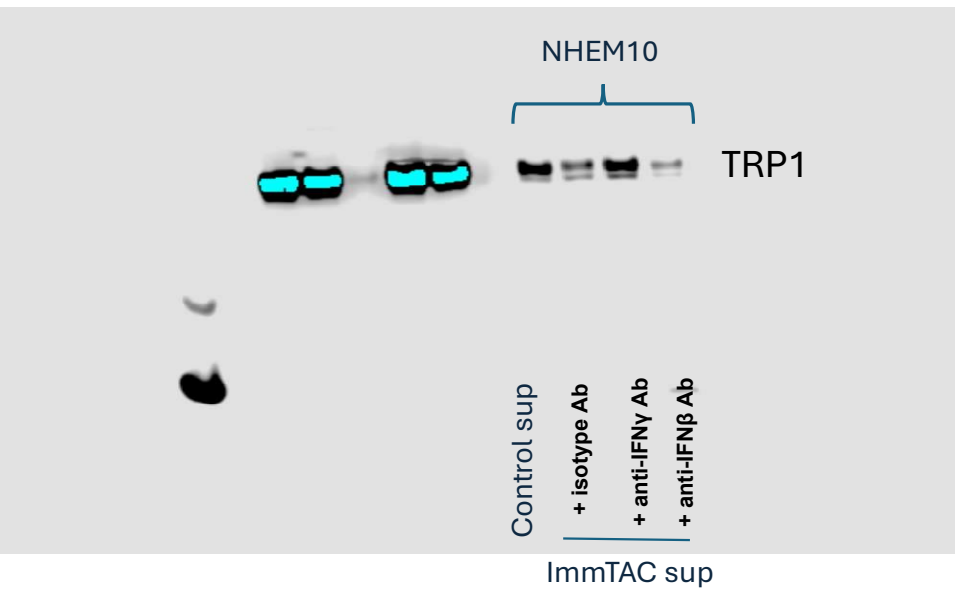

GAPDH

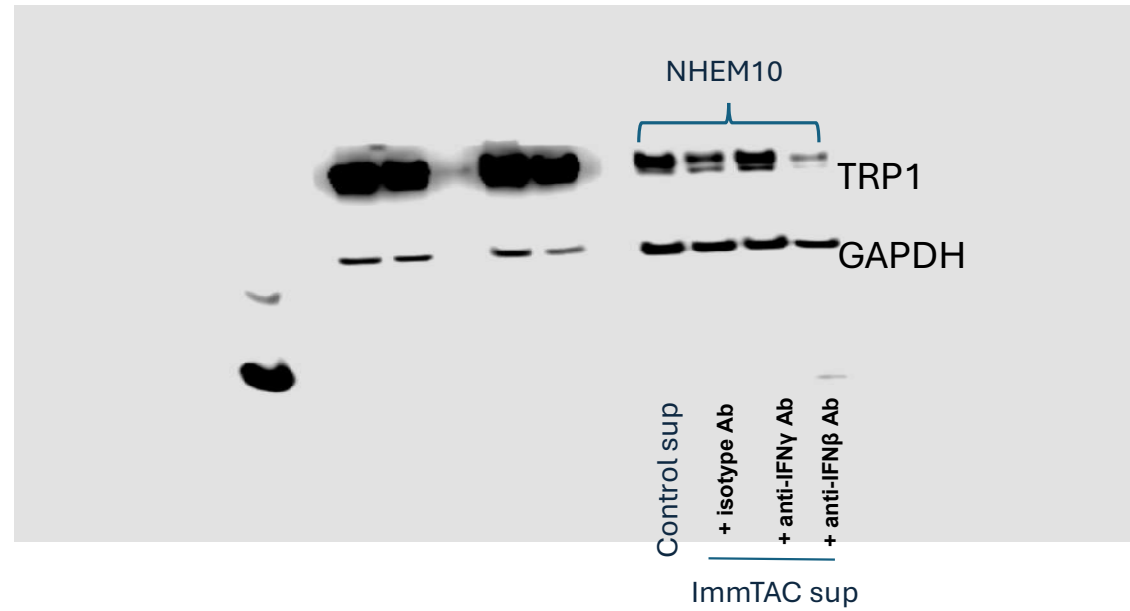

TRP2

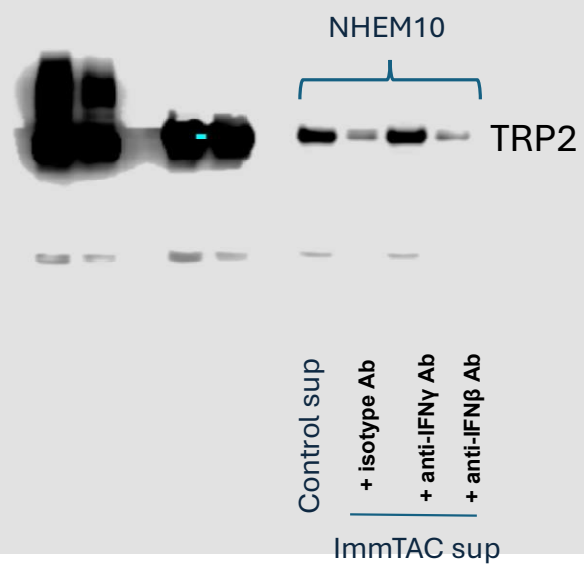

GAPDH

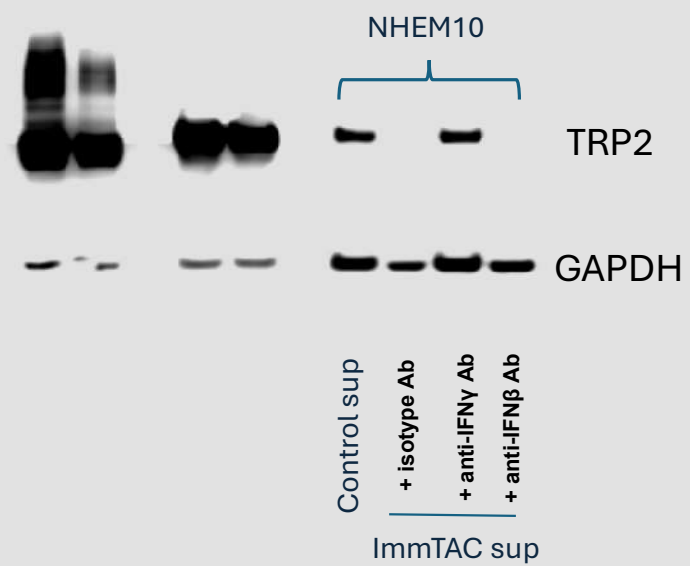

## MITF

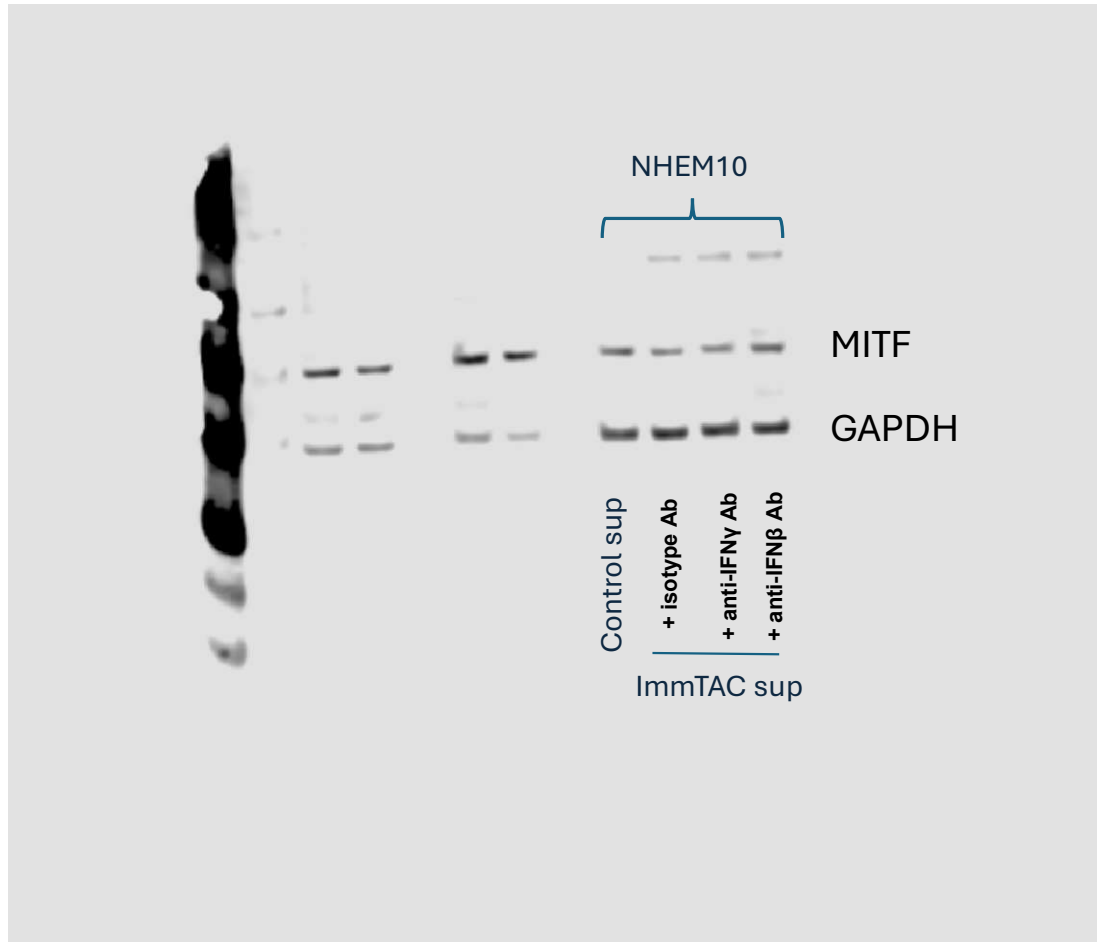

Supplement: Unedited blot and gel images [file jci-135-181464-s297.pdf]
